# Oxazolone-Induced Contact Hypersensitivity Reduces Lymphatic Drainage but Enhances the Induction of Adaptive Immunity

**DOI:** 10.1371/journal.pone.0099297

**Published:** 2014-06-09

**Authors:** David Aebischer, Ann-Helen Willrodt, Cornelia Halin

**Affiliations:** Institute of Pharmaceutical Sciences, Swiss Federal Institute of Technology, Zurich, Switzerland; Beth Israel Deaconess Medical Center, Harvard Medical School, United States of America

## Abstract

Contact hypersensitivity (CHS) induced by topical application of haptens is a commonly used model to study dermal inflammatory responses in mice. Several recent studies have indicated that CHS-induced skin inflammation triggers lymphangiogenesis but may negatively impact the immune-function of lymphatic vessels, namely fluid drainage and dendritic cell (DC) migration to draining lymph nodes (dLNs). On the other hand, haptens have been shown to exert immune-stimulatory activity by inducing DC maturation. In this study we investigated how the presence of pre-established CHS-induced skin inflammation affects the induction of adaptive immunity in dLNs. Using a mouse model of oxazolone-induced skin inflammation we observed that lymphatic drainage was reduced and DC migration from skin to dLNs was partially compromised. At the same time, a significantly stronger adaptive immune response towards ovalbumin (OVA) was induced when immunization had occurred in CHS-inflamed skin as compared to uninflamed control skin. In fact, immunization with sterile OVA in CHS-inflamed skin evoked a delayed-type hypersensitivity (DTH) response comparable to the one induced by conventional immunization with OVA and adjuvant in uninflamed skin. Striking phenotypic and functional differences were observed when comparing DCs from LNs draining uninflamed or CHS-inflamed skin. DCs from LNs draining CHS-inflamed skin expressed higher levels of co-stimulatory molecules and MHC molecules, produced higher levels of the interleukin-12/23 p40 subunit (IL-12/23-p40) and more potently induced T cell activation in vitro. Immunization experiments revealed that blockade of IL-12/23-p40 during the priming phase partially reverted the CHS-induced enhancement of the adaptive immune response. Collectively, our findings indicate that CHS-induced skin inflammation generates an overall immune-stimulatory milieu, which outweighs the potentially suppressive effect of reduced lymphatic vessel function.

## Introduction

Contact hypersensitivity (CHS) is an inflammatory reaction of the skin, which occurs upon exposure to haptens [1]–[3]. Haptens are low molecular weight chemical substances that can penetrate the skin and associate with endogenous proteins thereby generating strongly immunogenic hapten-protein complexes. Hapten exposure leads to the migration of activated Langerhans cells and dermal dendritic cells (DCs) via lymphatic vessels to draining lymph nodes (dLNs), where the hapten-protein complex is presented to naïve T cells. This leads to the generation of hapten-specific, skin-homing CD8^+^ and CD4^+^ T cells. It is known that the induction of hapten-specific T cells requires hapten-presentation on activated DCs, however the molecular mechanisms controlling DC activation by haptens are not completely understood. Exposure to haptens in the skin has been shown to induce the local release of damage-associated molecular pattern molecules (DAMPs), such as extracellular ATP or hyaluronic acid breakdown products. This is thought to lead to the activation of pattern-recognition receptors, resulting in the upregulation of co-stimulatory molecules and cytokine production by DCs [1], [4]–[6]. While the first encounter of a hapten (i.e. during the sensitization phase) typically remains asymptomatic, re-exposure to hapten in the skin (i.e. during the challenge phase) induces the activation of local hapten-specific T cells and the induction of a strong local inflammatory response [2], [3].

CHS-induced inflammation leads not only to the activation and dermal infiltration of leukocytes but also induces profound changes and remodeling in the stromal compartment, for example in the lymphatic vasculature [7]. Lymphatic vessels are essential for fluid drainage and additionally fulfill important immune functions by transporting leukocytes and lymph-borne antigen to draining lymph nodes (dLNs) [8]. Several groups including our own have shown that CHS-induced skin inflammation alters the lymphatic network and triggers a strong lymphangiogenic response in the inflamed skin and in dLNs [7], [9], [10]. In spite of this apparent expansion of the lymphatic network, recent studies indicate that the function of lymphatic vessels may be compromised in the context of CHS-induced skin inflammation. For example, oxazolone-induced CHS was shown to compromise lymphatic drainage [10], [11]. Similarly, one-time exposure (i.e. immune priming) to oxazolone transiently reduced lymphatic drainage and DC migration to dLNs [9]. Moreover, oxazolone-induced lymphatic dysfunction was recently shown to suppress T cell priming in dLNs and to reduce the severity of antigen-induced experimental autoimmune encephalomyelitis [12]. However, it has thus far not been determined whether CHS-induced changes in lymphatic function might compromise the induction of adaptive immunity in dLNs, in spite of the documented immune-stimulatory activity that haptens exert by enhancing DC maturation [1], [4], [5].

In this study we investigated how a pre-established acute or persistent CHS response impacts the induction of adaptive immunity towards a foreign antigen injected into the inflamed tissue. As a model we either induced and maintained a CHS response to oxazolone in the ear skin of wild-type (WT) mice, or alternatively established oxazolone-induced inflammation in the ear skin of hemizygous K14-VEGF-A-transgenic (K14-VEGF-A-tg) mice [13]. The latter cannot spontaneously down-regulate CHS-induced skin inflammation and develop chronic inflammatory skin lesions [7], [14]. Our analysis of lymphatic function in K14-VEGF-A-tg mice revealed that lymphatic drainage was significantly reduced and that DC migration was also partially compromised in the context of oxazolone-induced skin inflammation, depending on the time point analyzed. In spite of the reduction in lymphatic function, we observed in both CHS models that adaptive immunity to a foreign antigen was significantly enhanced in LNs draining inflamed skin, as assessed by the induction of a delayed-type hypersensitivity (DTH) response towards OVA and anti-OVA antibody production. Further analyses revealed that, in comparison to DCs present in resting LNs, DCs found in LNs draining inflamed skin expressed higher levels of IL-12/23p40 and displayed an increased capacity to activate T cells in vitro. Moreover, we showed that at the time point of T cell priming towards OVA, blockade of IL-12/23p40 partially reverted the CHS-induced enhancement of adaptive immunity. Overall, this study sheds new light on the mechanisms that regulate the induction of adaptive immune responses in the context of CHS-induced skin inflammation. It reveals that potentially immunosuppressive effects associated with reduced lymphatic function are outweighed by the overall immune stimulatory milieu that includes a mature DC phenotype.

## Results

### Acute and persistent skin inflammation reduces lymphatic drainage and differentially alters tissue cytokines and DC migration in K14-VEGF-A-tg mice

In our previous work describing CHS-induced changes in the lymphatic network [7] we made use of K14-VEGF-A-tg mice, in which murine vascular endothelial growth factor (VEGF)-A is constitutively expressed in the epidermis under the control of the keratinocyte-specific K14 promoter [13]. In contrast to WT mice, which typically clear a CHS-induced inflammatory response within a week [14], K14-VEGF-A-tg mice cannot down-regulate CHS-induced inflammation and develop persistent inflammatory skin lesions, which are characterized by epidermal hyperproliferation, leukocyte infiltration, and vascular remodeling [7], [14]–[16]. Persistence of inflammation is thought to be primarily caused by the strong angiogenic response apparent in the skin: VEGF-A overexpression leads to highly leaky and activated blood vessels, which favor edema formation. Moreover the blood vessels express high levels of adhesion molecules, such as E- and P-selectin, resulting in increased extravasation of inflammatory leukocytes [13], [14].

**Figure 1:**
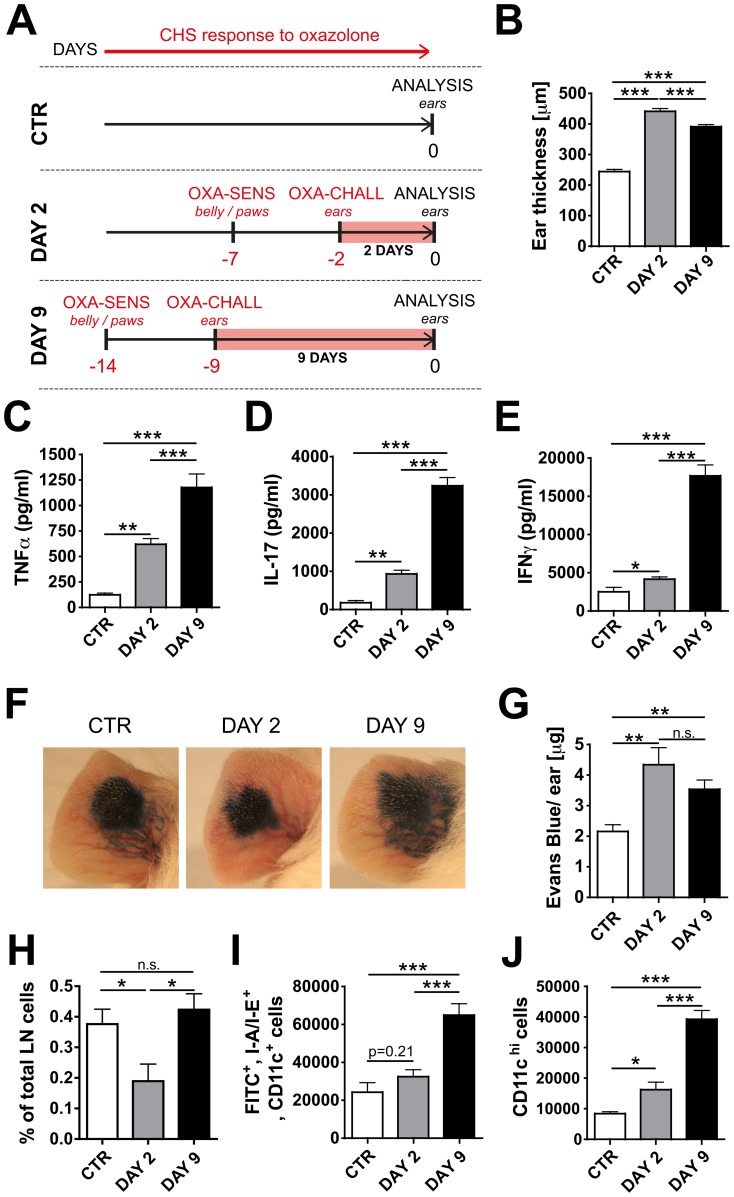
CHS-induced skin inflammation in K14-VEGF-A-tg mice differentially alters tissue cytokines, lymphatic drainage and DC migration at the DAY 2 and DAY 9 time point. (A) Schematic representation of the experiment: K14-VEGF-A-tg mice were grouped into a control (CTR) group, a DAY 2 and a DAY 9 group. On day -14 and day -7, mice from the DAY 9 group or the DAY 2 group, respectively, were sensitized by application of oxazolone (OXA) onto the belly and paws (indicated as OXA-SENS). 5 days later (day -9 or day -2), a CHS response was induced when challenging mice by topical oxazolone application onto the ears (indicated as OXA-CHALL). Ears of mice from all groups were analyzed on day 0. Notably, day 0 corresponded to 2 days (DAY 2 group) or 9 days (DAY 9 group) after challenge, i.e. the onset of inflammation (marked as a red bar). (**B**) The ear thickness was assessed as a measure of the ongoing inflammatory response. (**C-E**) ELISAs were performed on ear tissue protein extracts and the levels of the inflammatory cytokines (**C**) TNFα, (**D**) IFNγ and (**E**) IL-17 were measured. (**F,G**) To evaluate lymphatic drainage function, Evans Blue dye was injected into the ear skin of K14-VEGF-A-tg mice, and the dye content remaining in the ear was extracted and quantified 16 hours later. (**F**) Representative pictures taken immediately after Evans Blue injection. (**G**) Quantification revealed increased Evans Blue levels in the ears of mice with DAY 2- and DAY 9- inflamed ears, indicative of reduced drainage. (**H-J**) To study DC migration, FITC painting experiments were performed in the uninflamed (CTR), DAY 2- or DAY 9-inflamed ears. 18 hours after FITC application, mice were killed and single-cell suspensions of the ear draining auricular LNs were analyzed by FACS. (**H**) The percentage of I-A/I-E^+^CD11c^+^FITC^+^ cells in the dLN was significantly reduced in LNs draining DAY 2- but not DAY 9-inflamed ear skin. (**I**) Quantification of total cell numbers revealed that I-A/I-E^+^CD11c^+^FITC^+^ DCs were significantly increased in LNs draining DAY-9 but not DAY 2-inflamed ear skin. (**J**) FACS-based quantification or ear skin single –cell suspensions revealed that CD11c^hi^ DCs were significantly increased in DAY 2-and DAY 9-inflamed ear skin. Representative data from 1 out of 3 similar experiments (n = 5 mice/group) are shown. *p<0.05; **p<0.01; ***p<0.001.

Upon induction of a CHS response to oxazolone (**Figure 1A**) in the ear skin of K14-VEGF-A-tg mice inflammation persisted for at least nine days, as indicated by a strong ear swelling response (**Figure 1B**). Analysis of skin protein extracts revealed that the inflammatory cytokines TNFα, IFNγ and IL-17, were much more upregulated nine days as compared to two days after induction of inflammation (**Figure 1C–E**). Given the important role that lymphatic vessels play in DC migration and antigen drainage, we investigated the effect of inflammation on these two functions. To study drainage, Evans Blue was injected into the ear pinna [11], [17]. Five minutes later lymphatic vessels in uninflamed ears were readily draining Evans Blue, but drainage appeared to be reduced in the ears of mice with acute (DAY 2) inflammation (**Figure 1F**). By contrast, in ears with persistent (DAY 9) inflammation, Evans Blue was taken up into dilated and apparently leaky vessels (**Figure 1F**). Quantification performed 16 hours later revealed that at both time points significantly more dye was retained in inflamed as compared to uninflamed ears (**Figure 1G**), indicative of a reduced lymphatic drainage function. Next we performed fluorescein isothiocyanate (FITC) painting experiments [18] to investigate how DC migration to dLNs was modulated in the context of oxazolone-induced skin inflammation. Eighteen hours after skin painting, mice were sacrificed and the number of FITC^+^I-A/I-E^+^CD11c^+^ DCs was quantified in dLNs by flow cytometry. This analysis revealed that the percentage of FITC^+^I-A/I-E^+^CD11c^+^ cells amongst all nodal cells was significantly reduced in LNs draining DAY 2-inflamed skin but returned to control levels in LNs draining DAY 9-inflamed skin (**Figure 1H**). When quantifying total numbers of FITC^+^I-A/I-E^+^CD11c^+^ cells in dLNs, a significant increase in DC accumulation over control levels was only detected at the DAY 9, but not a the DAY 2 time point (**Figure 1I**). FACS analysis performed on ear skin single-cell suspensions revealed a significant increase in CD11c^+^ DC numbers in DAY 2- and DAY 9-inflamed ear skin, as compared to uninflamed control ear skin (**Figure 1J**). Since the increase in the accumulation of FITC^+^ DCs in dLNs at the DAY 9 time point (**Figure 1I**) was proportional to the increase in total DC numbers present in DAY 9-inflamed skin (**Figure 1J**), these data indicated that DC migration was not enhanced at this time point. Similar results were also obtained when performing in vitro DC emigration experiments with control and DAY 2- or DAY 9-inflamed ears (**Figure S1 in File S1**). In this setup, the total number and the percentage of DCs that emigrated overnight from acutely inflamed (DAY 2) ear explants into the culture medium was profoundly reduced in comparison to the number and percentage of DCs emigrating from uninflamed ears. Interestingly, also this defect in DC emigration was transient, since a similar percentage of DCs emigrated from DAY 9-inflamed ears as compared to control ears (**Figure S1 in File S1**).

Overall, skin inflammation in K14-VEGF-A-tg mice lead to a reduction in lymphatic drainage and differentially affected tissue cytokine levels and DC migration in dLNs, depending on the time-point analyzed. These clear differences prompted us to include both the DAY 2 and DAY 9 time points in our further experiments.

### CHS-induced skin inflammation facilitates the induction of adaptive immunity in dLNs of K14-VEGF-A tg-mice

**Figure 2:**
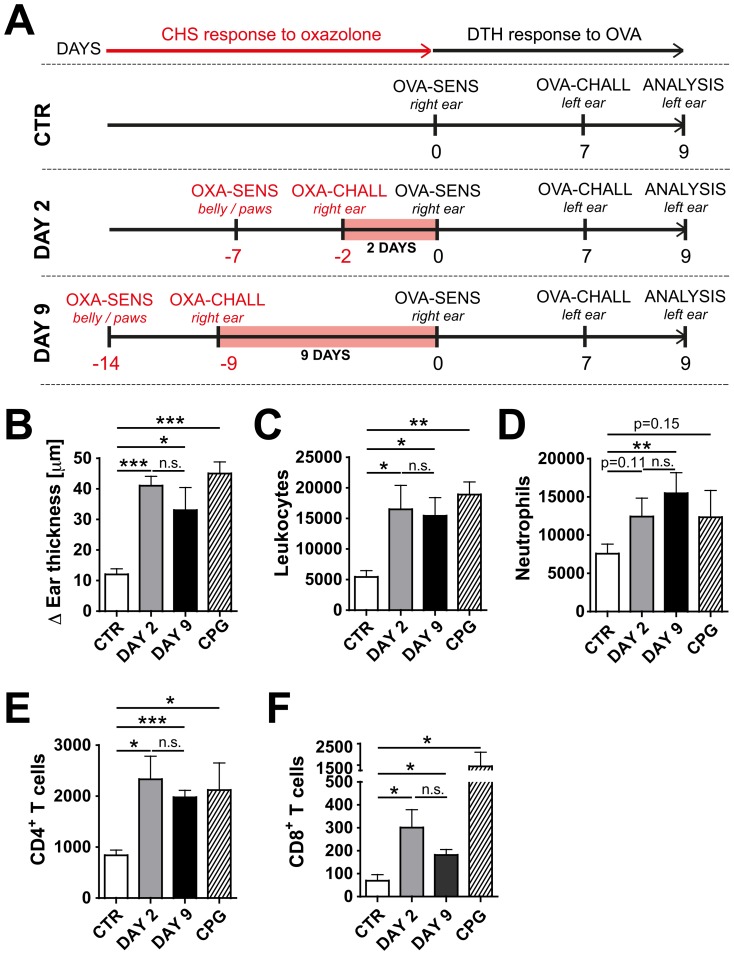
Skin inflammation enhances the induction of a DTH response to OVA in K14-VEGF-A-tg mice. (A) Schematic representation of the experiment: K14-VEGF-A-tg mice were grouped into a control (CTR) group, a DAY 2 and a DAY 9 group. On day -14 and day -7, mice from the DAY 9 group or the DAY 2 group, respectively, were sensitized by application of oxazolone (OXA) onto the belly and paws (indicated as OXA-SENS). 5 days later (day -9 or day -2), a CHS response was induced by challenging mice with oxazolone on the right ear (indicated as OXA-CHALL). On day 0, corresponding to 2 days (DAY 2 group) or 9 days (DAY 9 group) after the onset of inflammation (marked as a red bar), a DTH experiment was initiated in mice from all treatment groups. To this end, all mice were immunized by OVA injection into the CHS-inflamed or control right ear (indicated as OVA-SENS). On day 7, a DTH response to OVA was induced by OVA injection into the left ear (indicated as OVA-CHALL). The strength of the immune response in the left ear was analyzed two days later (day 9). (**B**) The ear swelling response in the left ear was significantly stronger in mice that had been immunized by OVA injection into the inflamed, right ear. The Δ ear thickness is shown, defined as the difference between the ear thickness measured at baseline and the ear thickness measured two days after DTH induction. CPG group: As a positive control group, some untreated control mice were immunized by injection of OVA plus CpG into their uninflamed right ears (R) on day 0, followed by a regular OVA challenge into the left ear (L) on day 7 (as described in 2A). (**C–F**) FACS analysis was performed on single-cell suspensions of the DTH-challenged left ear (L) to quantify leukocyte infiltration. Quantification of (**C**) total leukocytes (CD45^+^), (**D**) neutrophils (CD11b^+^.Gr1^+^), (**E**) CD4^+^CD3^+^CD45^+^ T cells and (**F**) CD8^+^CD3^+^CD45^+^ T cells. Representative data from 1 out of 3 similar experiments (n = 5 mice/group) are shown. *p<0.05; **p<0.01; ***p<0.001.

To analyze the effect of DAY 2 and DAY 9 skin inflammation on the induction of adaptive immunity, a classical DTH experiment [19] was performed. To this end, K14-VEGF-A-tg mice were sensitized by injection of sterile OVA into their uninflamed or DAY 2- or DAY 9-inflamed right ears (**Figure 2A**). As a positive immunization control, some untreated K14-VEGF-A-tg mice were sensitized by injection of OVA in combination with a TLR9 agonist, i.e. a CpG-containing oligonucleotide, into the uninflamed right ear. After seven days all mice were challenged by OVA injection into the contralateral, uninflamed left ear. Forty eight hours later the thickness of the challenged left ear was measured as an indication of the strength of the DTH response induced (**Figure 2B**). As expected, a strong DTH-induced ear swelling was observed in mice immunized by OVA/CpG injection into their uninflamed right ears (**Figure 2B**). Virtually no ear swelling developed in mice sensitized by injection of sterile OVA into uninflamed ear skin (**Figure 2B**), whereas mice that had been sensitized by OVA injection into DAY 2- or DAY 9-inflamed ears, mounted an ear swelling response that was comparable to the one observed in the OVA/CpG group (**Figure 2B**). Furthermore, total leukocyte (**Figure 2C** **and Figure S2A in File S1**) and CD4^+^ and CD8^+^ T cell (**Figure 2E–F** **and Figure S2C in File S1**) recruitment into the DTH-challenged, left ear was similarly enhanced in these groups as in the OVA/CpG group. Moreover, neutrophil recruitment was enhanced, but this was only significant at the DAY 9 time point (**Figure 2D** **and Figure S2B in File S1**). Similar results were obtained with an additional model of adaptive immune induction, namely, a CHS response to FITC (**Figure S3 in File S1**). Thus, using two different immunization models (**Figure 2** **, Figure S3 in File S1**) we observed that the induction of an adaptive immune response in K14-VEGF-A-tg mice was strikingly facilitated in presence of CHS-induced skin inflammation.

### The induction of adaptive immune responses is also enhanced in LNs draining inflamed skin of WT mice

**Figure 3:**
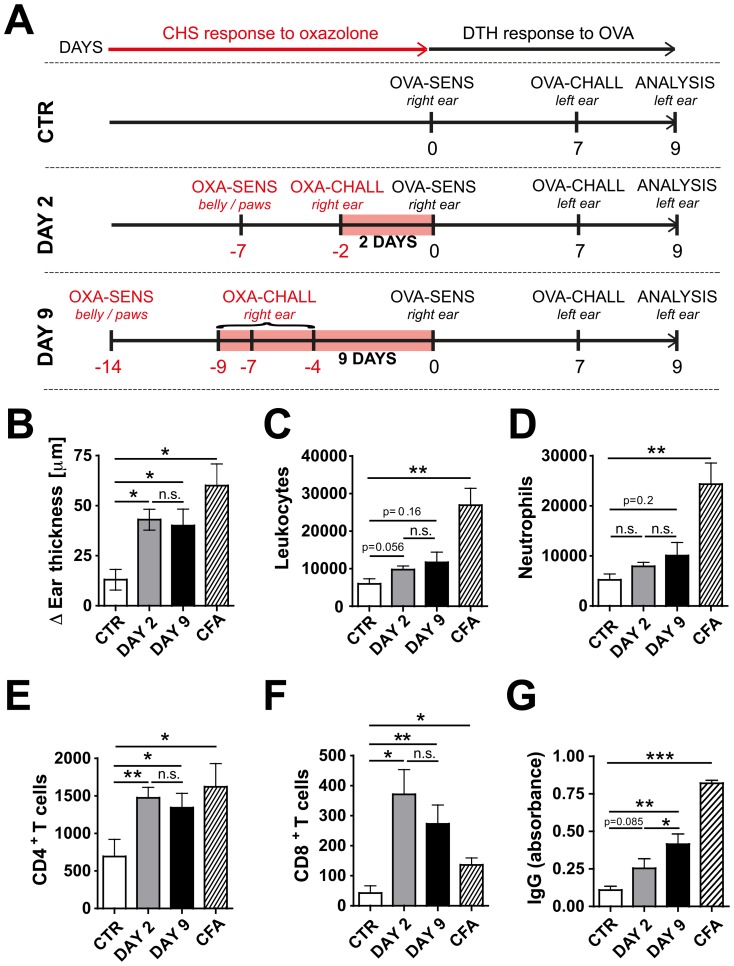
Skin inflammation enhances the induction of a DTH response to OVA in WT mice. (A) Schematic representation of the experiment: WT mice were grouped into a control (CTR) group, a DAY 2 and a DAY 9 group. On day -14 and day -7, mice from the DAY 9 group or the DAY 2 group, respectively, were sensitized by application of oxazolone (OXA) onto the belly and paws (indicated as OXA-SENS). 5 days later (day -9 or day -2) a CHS response was induced, by challenging mice from the DAY 9 or DAY 2 groups with oxazolone on the right ear (indicated as OXA-CHALL). In mice belonging to the DAY 9 group, OXA was repeatedly applied onto the right ear on day -7 and day -4, to maintain the inflammatory response. On day 0, corresponding to 2 days (DAY 2 group) and 9 days (DAY 9 group) after the onset of inflammation (marked as a red bar), a DTH experiment was initiated in mice from all treatment groups. To this end, all mice were immunized by OVA injection into the CHS-inflamed or control right ear (indicated as OVA-SENS). On day 7, a DTH response to OVA was induced by OVA injection into the left ear (indicated as OVA-CHALL). The strength of the immune response in the left ear was analyzed two days later (day 9). (**B**) The ear swelling response in the left ear was significantly stronger when mice had been immunized by OVA injection into the inflamed, right ear. The Δ ear thickness is shown, defined as the difference between the ear thickness measured at baseline and the ear thickness measured two days after DTH induction. CFA group: As a positive control group, some untreated mice were immunized by injection of OVA plus CFA into the uninflamed right ear (R) on day 0, followed by a regular OVA challenge into the left ear (L) on day 7 (as described in 3B). (**C–F**) FACS analysis was performed on single-cell suspensions of the DTH-challenged, left ear to quantify leukocyte infiltration. Quantification of (**C**) total leukocytes (CD45^+^), (**D**) neutrophils (CD11b^+^Gr1^+^), (**E**) CD4^+^CD3^+^CD45^+^ T cells and (**F**) CD8^+^CD3^+^CD45^+^ T cells. (**G**) Quantification of OVA-specific total IgG in the serum of mice. Representative data from 1 out of 3 similar experiments (n = 6–7 mice/group) are shown. *p<0.05; **p<0.01.

Thus far, our experiments showed that the presence of pre-established CHS-induced skin inflammation facilitated the induction of adaptive immune responses in dLNs of K14-VEGF-A-tg mice. However, various studies have recently reported that VEGF-A may directly or indirectly affect T cell priming [20]–[23]. To confirm our results in a VEGF-A-independent model, similar experiments were performed in WT mice. In analogy to our experiments in K14-VEGF-A-tg mice, WT mice were sensitized and subsequently challenged with oxazolone. To establish persistent inflammation, corresponding to the DAY 9 time point analyzed in the K14-VEGF-A-tg mouse model (**Figure 1A**), WT mice were re-challenged with oxazolone on day 2 and day 5 (**Figure 3A**). Treatment resulted in a strong ear swelling response at both the DAY 2 and DAY 9 time points analyzed (data not shown). In analogy to the experiments performed in the K14-VEGF-A-tg mouse model, a DTH response was subsequently induced by injection of OVA into uninflamed control or DAY 2- or DAY 9-inflamed right ears, followed by challenge with OVA into the contralateral ears seven days later (**Figure 3A**). As a positive control, a DTH response was also induced in completely untreated WT mice, which were immunized by injection of OVA emulsified in Complete Freund's Adjuvant (CFA). In agreement with our observations made in the K14-VEGF-A-tg mouse model (**Figure 2**), DTH-induced ear swelling was much more pronounced when immunization with OVA had occurred in inflamed ear skin as compared to uninflamed ear skin of WT mice (**Figure 3B**). Again, the DTH response was almost as strong as the one induced in the OVA/CFA immunization control group (**Figure 3B**). Total leukocyte and neutrophil infiltration into the OVA-challenged ears appeared to be slightly increased in the DAY 2 and DAY 9-inflamed groups, but this did not reach statistical significance (**Figure 3C,D**). By contrast, the numbers of CD4^+^ and CD8^+^ T cells recovered from OVA-challenged ears were significantly increased when sensitization to OVA had occurred in DAY 2- or DAY 9-inflamed ears, as compared to uninflamed control ears (**Figure 3E,F**). Moreover, injection of OVA into DAY 9-inflamed skin favored the induction of adaptive B cell responses, as revealed by ELISA-based quantification of OVA-specific total IgG in serum (**Figure 3G**). Similar results were obtained when quantifying OVA-specific IgG_1_ and IgG_2a_ (data not shown). Taken together, CHS-induced skin inflammation similarly facilitated the induction of adaptive immunity in K14-VEGF-A-tg mice and in WT mice, indicating that these findings were independent of VEGF-A transgene expression and are of more general significance.

### DCs present in LNs draining CHS-inflamed skin of WT or K14-VEGF-A-tg mice are more potent inducers of T cell activation in vitro

**Figure 4:**
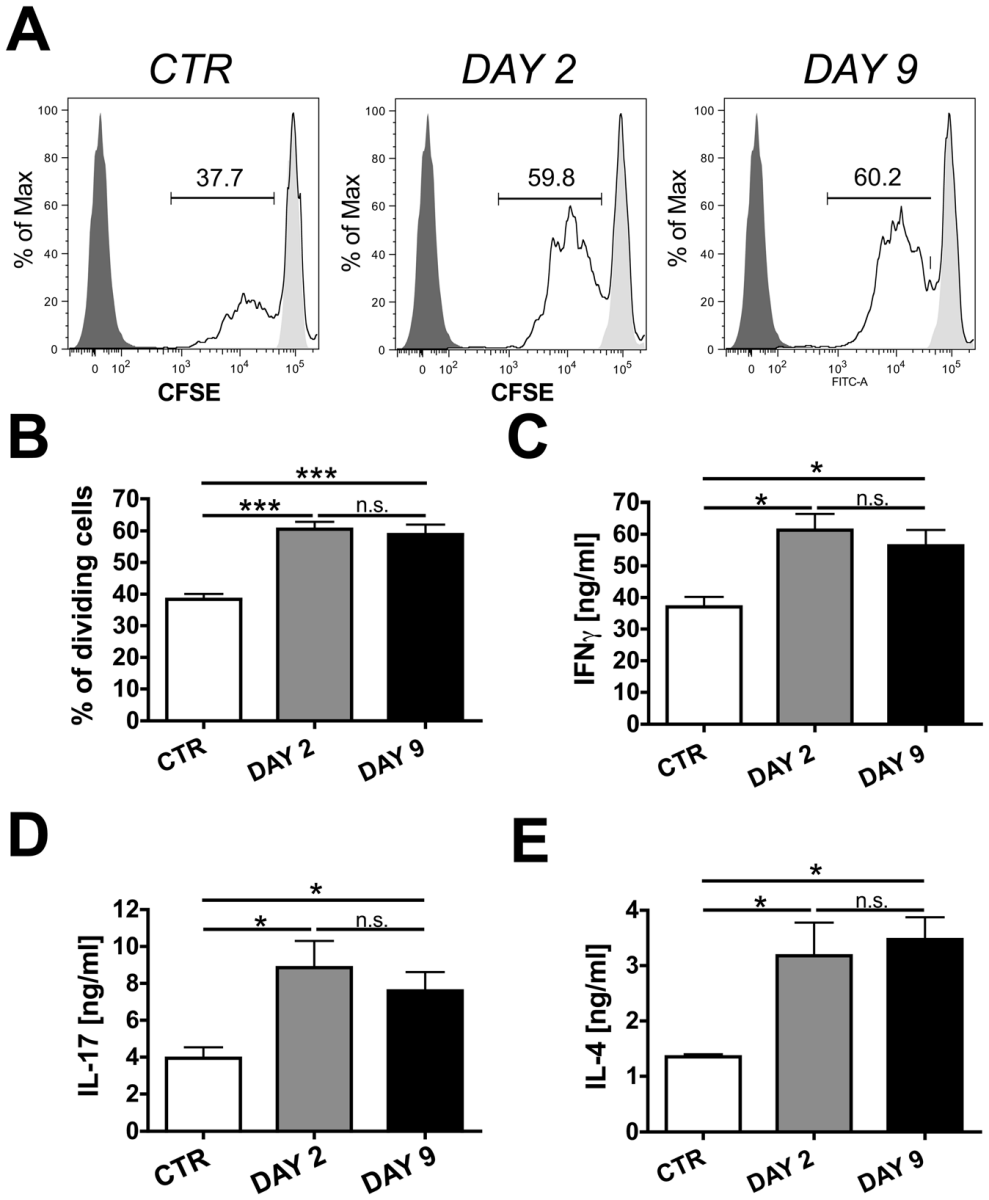
DCs isolated from LNs draining CHS-inflamed skin of WT mice are more potent inducers of T-cell activation in vitro. CD11c^+^ DCs were FACS-sorted from the auricular LNs of WT BALB/c mice with uninflamed (CTR) or DAY 2- or DAY 9-inflamed ear skin. Subsequently, CFSE-labeled TCR transgenic CD4^+^ T cells isolated from DO11.10 mice were incubated for 3 days with DCs and OVA peptide at a T cell: DC ratio of 5:1. CFSE dilution assays revealed that DCs isolated from LNs draining DAY 2- and DAY 9-inflamed skin were significantly more potent in inducing T cell proliferation than DCs isolated from control LNs. (**A**) Representative FACS plots showing CFSE dilution in dividing T cells. Numbers indicate the percentage of gated cells. (**B**) Quantification of the percentage of proliferating T cells. Significantly higher levels of (**C**) IFNγ, (**D**) IL-17 and (**E**) IL-4 were quantified in the supernatant of co-cultures containing DCs isolated from LNs draining DAY 2- or DAY 9-inflamed skin. Representative data from 1 out of 2 similar experiments (n = 3) per condition are shown. *p<0.05.

Induction of adaptive immune responses in dLNs not only depends on lymphatic drainage and DC migration (**Figure 1**), but also on the phenotype of the antigen-presenting DCs [24]. We next assessed the capacity of DCs isolated from LNs draining uninflamed or CHS-inflamed ear skin (DAY 2 and DAY 9) of WT mice to induce T cell proliferation in vitro. To this end, CD11c^+^ I-A/I-E^+^ DCs were FACS-sorted from dLNs and co-cultured in the presence of OVA-peptide with cognate CFSE labeled T cell receptor (TCR)-transgenic CD4^+^ T cells, which had been isolated from the spleen and LNs of DO11.10 mice [25]. FACS analysis performed three days later revealed that DCs from LNs draining DAY 2- or DAY 9-inflamed skin were more potent inducers of T cell proliferation, as compared to DCs isolated from LNs draining uninflamed control skin (**Figure 4A,B**). Moreover, the supernatant of co-cultures containing DCs isolated from LNs draining DAY 2- or DAY 9-inflamed skin contained significantly higher levels of the effector cytokines IFNγ, IL-17 and IL-4 (**Figure 4C–E**). Almost identical results were obtained when performing these experiments with DCs isolated from LNs draining uninflamed or CHS-inflamed ear skin of K14-VEGF-A-tg mice (**Figure S4 in File S1**).

### IL-12/23p40 is consistently upregulated in DCs in LNs draining CHS-inflamed skin of WT or K14-VEGF-A-tg mice

**Figure 5:**
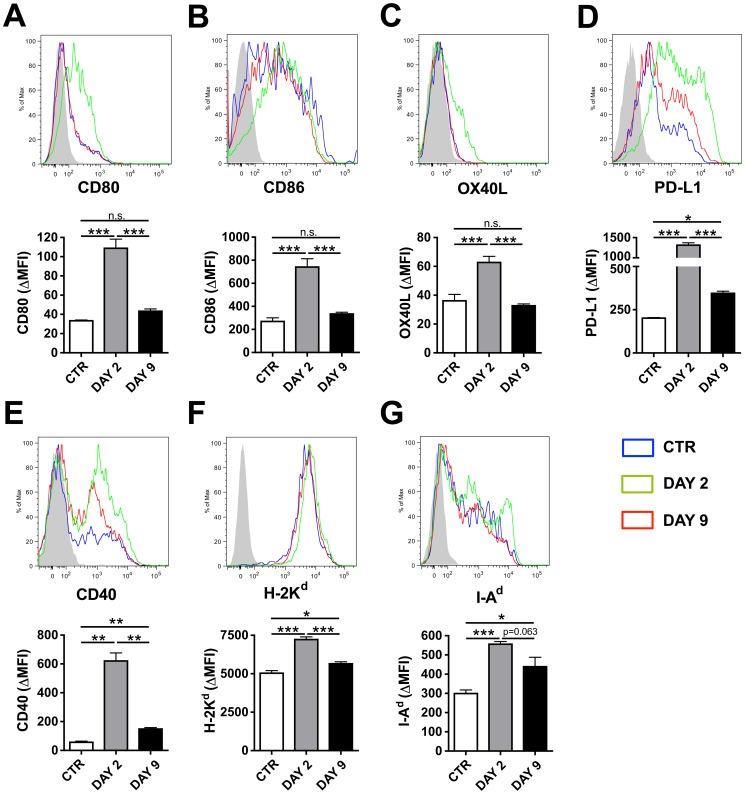
DCs in LNs draining CHS-inflamed skin in WT mice display changes in their expression of co-stimulatory and MHC molecules. FACS analysis was performed on CD11c^+^ DCs present in auricular LNs draining DAY 2- and DAY 9-inflamed or uninflamed control (CTR) skin of WT mice. (**A–G**) Analysis of the expression levels of co-stimulatory and MHC molecules. The upper panel shows representative FACS plots. Blue line: CTR; green line: DAY 2; red line: Day 9; filled histogram: isotype control. To reduce complexity, only one out of three similar isotype control stainings is shown. The lower graph shows the Δ MFI (defined as the MFI of the specific staining – the MFI of the isotype control staining) values measured for each condition (n = 3-4 mice/group). (**A**) CD80, (**B**) CD86, (**C**) OX40L, (**D**) PD-L1, (**E**) CD40, (**F**) H-2K^d^ and (**G**) I-A^d^. Representative data from 1 out of 2 similar experiments are shown. *p<0.05; **p<0.01; ***p<0.001.

To investigate the molecular differences between DCs in LNs draining CHS-inflamed as compared to control skin, FACS analysis was performed on LN single-cell suspensions and the DC phenotype analyzed by gating on CD11c^+^ cells. This analysis revealed that the co-stimulatory molecules CD80, CD86, OX40L, PD-L1, CD40 and MHC class I (H-2K^d^) and class II (I-A^d^) molecules were strikingly upregulated at the DAY 2 time point (**Figure 5A-G**). By contrast, at the DAY 9 time point, a significant yet less strong induction was only observed for PD-L1, CD40, H-2K^d^ and I-A^d^ (**Figure 5D–G**), whereas no induction of CD80, CD86, OX40L over the levels found in control DCs was detected (**Figure 5A–C**). Again, very similar findings were made when analyzing the DC phenotype in the K14-VEGF-A-tg mouse model (**Figure 5A–G**). The exception being that in this model CD40 was equally but only slightly upregulated in the context of both DAY 2- and DAY 9-CHS (**Figure S5E in File S1**). DCs in LNs can be divided into different populations based on their function and migratory properties. A commonly used approach to distinguish between lymph-borne DCs, which arrive in dLNs via afferent lymphatics, and blood-borne DCs, which arrive via the blood circulation, is based on differences in MHCII expression levels [26]–[28]. In order to determine whether the changes that we observed in co-stimulatory molecule expression (**Figure 5**) applied to both populations, the same analysis was performed but this time distinguishing between CD11c^+^ I-A/I-E^hi^ and CD11c^+^I-A/I-E^low/int^ cells (**Figure S6 in File S1**). Although basal expression levels of CD80, CD86, OX40L, PD-L1, CD40 and H-2K^d^ and I-A^d^ differed between the two populations, the general response pattern to CHS-induced skin inflammation was similar in CD11c^+^ I-A/I-E^hi^ cells and in CD11c^+^I-A/I-E^low/int^ cells (**Figure S6 in File S1**). Notably, the relative composition of migratory versus LN-resident DCs only marginally changed in the context of skin inflammation (**Figure S7 in File S1**).

**Figure 6:**
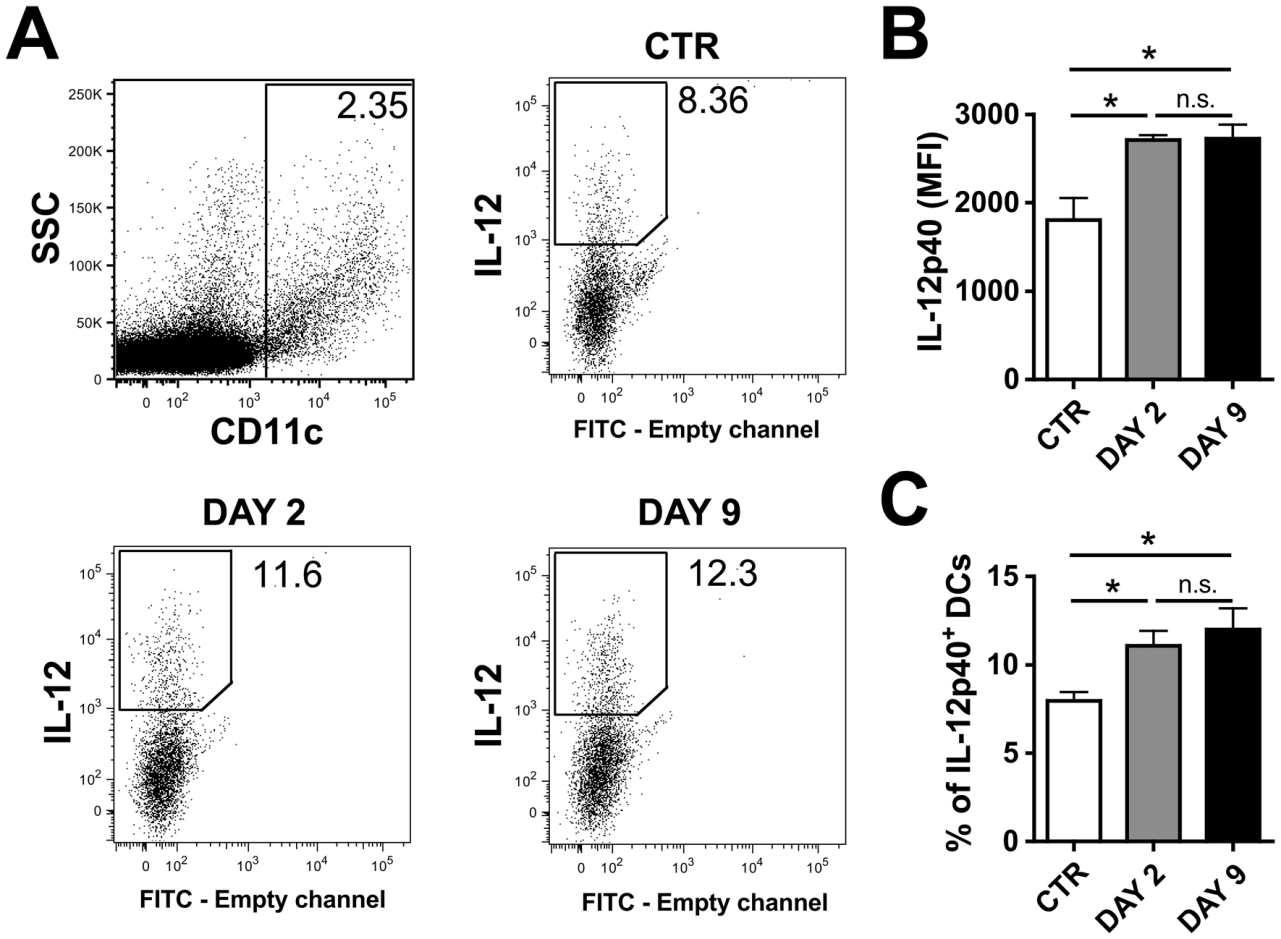
DCs in LNs draining sites of skin inflammation in WT mice upregulate IL-12/23p40 expression. Intracellular FACS analysis of IL-12/23-p40 in CD11c^+^ DCs present in LNs draining uninflamed control (CTR) or DAY 2- or DAY 9-inflamed ear skin of WT mice. (**A**) Representative FACS plots of the different groups are shown. (**B**) The MFI of IL-12/23-p40 staining on CD11c^+^ DCs was significantly higher in DCs present in LNs draining DAY 2- and DAY 9-inflamed ear skin as compared to LNs draining CTR skin. (**C**) Furthermore, a greater percentage of DCs in inflammation-dLNs stained positive for IL-12/23-p40 expression as compared to DCs in control LNs. Representative data from 1 out of 2 similar experiment (n = 3 mice/group) are shown. *p<0.05.

FACS analysis was performed to investigate intracellular expression of IL-12/23p40, a subunit shared by both IL-12 and IL-23 [29]. The mean fluorescence intensity (MFI) and the percentage of IL-12/23p40-expressing cells was consistently higher in DCs in LNs draining DAY 2- and DAY 9-inflamed skin as compared to DCs in LNs draining control skin. Similar findings were seen in both WT (**Figure 6A–C**) and K14-VEGF-A-tg mice (**Figure S8A–C in File S1**).

In conclusion, in both mouse models investigated, MHC and co-stimulatory molecules - with the exception of CD40 - were differentially induced in DCs at the two time points analyzed (**Figure 5A–G**, **Figure S5A–G in File S1**). Since an inflammation-induced enhancement of adaptive immunity was observed in both DAY 2- and DAY 9-inflamed skin (**Figure 2** **&** **3**), the differential expression pattern of these molecules made them less likely and less straight-forward candidates for the observed CHS-induced immune-enhancement. In contrast, IL-12/23p40 was consistently upregulated at both the DAY 2 and DAY 9 time points analyzed and in both models investigated (**Figure 6A–C**, **Figure S8A–C in File S1**), indicating a potential contribution of this molecule.

### Blockade of IL-12/23p40 reduces the inflammation-induced increase in adaptive immune responses in WT mice

**Figure 7:**
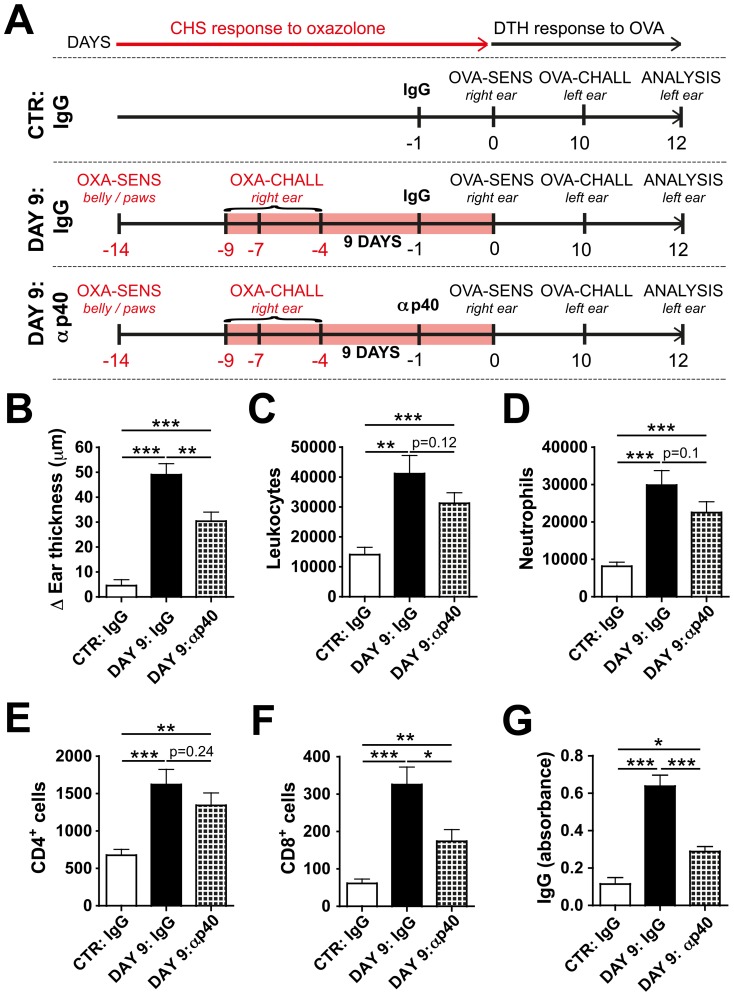
Blockade of IL-12/23-p40 reduces the induction of adaptive immune responses towards OVA in WT mice with DAY 9-inflamed ears. (A) Schematic representation of the experiment: WT mice were grouped into a control (CTR): IgG group, a DAY 9: IgG or a DAY 9: αp40 group. On day -14 mice in the DAY 9: IgG and DAY 9:αp40 groups were sensitized by application of oxazolone (OXA) onto the belly and paws (indicated as OXA-SENS). 5 days later (day -9) a CHS response was induced, by challenging mice with oxazolone on the right ear (indicated as OXA-CHALL). OXA was repeatedly applied onto the right ear on day -7 and day -4, to maintain the inflammatory response. On day -1 the DAY 9:αp40 group was treated with an IL-12/23-p40 blocking antibody (αp40). Mice in the DAY 9: IgG and the CTR: IgG groups were treated with an IgG control antibody (IgG). On day 0 a DTH experiment was initiated in mice from all treatment groups. To this end, all mice were immunized by OVA injection into the CHS-inflamed or control right ear (indicated as OVA-SENS). On day 10, a DTH response to OVA was induced by OVA injection into the left ear (indicated as OVA-CHALL). The strength of the immune response in the left ear was analyzed two days later (day 12). (B) The ear swelling response in the left ear was significantly weaker when mice had been treated with the anti-IL-12/23p40 antibody compared to mice treated with the corresponding isotype control. The Δ Ear thickness is shown, which is defined as the difference between the ear thickness measured at baseline and the ear thickness measured two days after DTH induction. FACS analysis was performed on single-cell suspensions of the DTH-challenged, left ear to quantify leukocyte infiltration. Quantification of (C) total leukocytes (CD45^+^), (D) neutrophils (CD11b^+^Gr1^+^ cells), (E) CD4^+^CD3^+^CD45^+^ T cells and (F) CD8^+^CD3^+^CD45^+^ T cells. (G) Quantification of serum OVA-specific IgG titer. Data from 2 pooled experiments (n = 12–14 mice/group) are shown. *p<0.05; **p<0.01; ***p<0.001.

To investigate whether induction of IL-12/23-p40 accounted for the inflammation-induced enhancement of adaptive immunity, WT mice with persistent (DAY 9) skin inflammation in their right ears (as described in **Figure 3A**) were treated with an IL-12/23-p40 blocking or control antibody one day prior to immunization with OVA into the inflamed right ear (**Figure 7A**). Ten days later - a time-point at which 95% of the IL-12/23-p40-blocking antibody had been cleared from circulation (data not shown) – mice were challenged by injection of OVA into the uninflamed left ear. As in previous experiments (**Figure 2B** **,** **Figure 3B**), the DTH response to OVA was strikingly increased when OVA immunization had occurred in inflamed skin (**Figure 7B**). However, in mice that had been treated with anti-IL-12/23-p40 prior to OVA immunization, DTH-induced ear swelling was significantly reduced (**Figure 7B**). Moreover, a tendency towards reduced leukocyte infiltration into the ears of mice treated with anti-IL-12/23-p40 was observed (**Figure 7C-F**), but this only reached statistical significance in the case of CD8^+^ T cells (**Figure 7F**). At the same time, anti-OVA antibody levels in serum were significantly reduced in anti-IL-12/23-p40-treated mice (**Figure 7G**). Overall, we conclude that blockade of IL-12/23-p40 during the afferent phase of the immune response partially reverted the CHS-induced enhancement of adaptive immunity observed in this model.

## Discussion

In this study we have analyzed how the presence of pre-established skin inflammation induced by CHS response to oxazolone affects the induction of adaptive immunity. Several recent studies have shown that topical application of oxazolone and oxazolone-induced CHS compromise the functionality of the lymphatic network. Experiments performed in WT [10] or in K14-VEGF-A-tg mice [11] demonstrated that lymphatic drainage is reduced in the context of oxazolone-induced CHS, or even upon single exposure of skin to oxazolone [9], [12]. In agreement with these studies we observed that in K14-VEGF-A-tg mice lymphatic drainage function was consistently reduced during both acute (DAY 2) and persistent (DAY 9) oxazolone-induced skin inflammation (**Figure 1G**). Interestingly, the images taken immediately after Evans Blue injection (**Figure 1F**) suggested that drainage might have been compromised for different reasons at the two time points analyzed. While on DAY 2 the uptake into lymphatic vessels was strikingly reduced, the dye was taken up into dilated but apparently leaky lymphatic vessels on DAY 9 (**Figure 1F**). The exact reasons for these differences are unclear, but they could be associated with time-dependent changes in tissue inflammation (**Figure 1C–E**) and in inflammation-induced lymphangiogenic remodeling [7], [14]. With regards to the functionality of DC migration, the results obtained in our study were less clear. Our combined results from FITC painting experiments (**Figure 1H–J**) and ear emigration experiments (**Figure S1 in File S1**) performed in K14-VEGF-A-tg mice indicated that the capacity of DCs to migrate was reduced in the context of DAY 2-inflammation but recovered to baseline levels at the DAY 9 time point. These temporal differences in the DC migratory response are similar to data from a previous study reporting that a single topical exposure to oxazolone induced a profound but transient decrease in DC migration in WT mice [9]. Overall, we observed that CHS-induced skin inflammation in K14-VEGF-A-tg mice compromised lymphatic drainage and transiently reduced the capacity of DCs to migrate to dLNs. However, in spite of these apparent deficiencies in lymphatic function, the induction of adaptive immunity towards OVA was more potent when immunization was induced in CHS-inflamed as compared to control skin (**Figure 2** **,** **Figure 3**). In fact, the injection of OVA alone into pre-inflamed skin resulted in a similar degree of adaptive immune induction as when OVA and an adjuvant were simultaneously injected into uninflamed skin. These findings indicate that the overall immune-stimulatory environment created by CHS-induced tissue inflammation outweighed the potentially immunosuppressive effects associated with reduced lymphatic function.

These findings were consistently made in two different mouse models of CHS, namely during oxazolone-induced skin inflammation in WT mice and in K14-VEGF-A-tg mice (**Figure 2** **,** **Figure 3**). The advantage of the latter model is that sensitization and challenge with oxazolone establishes non-resolving skin inflammation, which results in the opportunity to study differences in lymphatic function and immune induction at early (DAY 2) and later (DAY 9) time points without the need of repeatedly exposing the skin to oxazolone. Our previous work has shown that in both models the lymphatic network in the skin and in dLNs undergoes a strong proliferative expansion within the first nine days after onset of inflammation [7]. A limitation of the K14-VEGF-A-tg mouse model might be its potential bias towards generating VEGF-A-mediated effects that might not accompany inflammatory responses in WT mice. In fact, abundant literature has recently implicated VEGF-A in various aspects of adaptive immunity, such as DC maturation and T helper cell polarization [20]–[23]. However, a comparison of the results obtained in the WT and in the K14-VEGF-A-tg model revealed that they were remarkably similar (**Figure 2** **,** **Figure 3**). Also the functional and phenotypic analysis of DCs isolated from LNs draining uninflamed or CHS-inflamed skin detected no major differences between DCs from WT and DCs from K14-VEGF-A-tg mice (**Figures 4** **–** **6** **, Figures S4,5&7 in File S1**). One exception was CD40 expression, which displayed higher baseline levels in control DCs from K14-VEGF-A-tg mice (**Figure 5G** **, Figure S5G in File S1**). Moreover, CD40 was strikingly upregulated at the DAY 2 time point in DCs from WT but not in DCs from K14-VEGF-A-tg mice (**Figure 5E** **, Figure S5E in File S1**). Interestingly, we have recently reported that VEGF-A is strongly upregulated in epidermal keratinocytes in the context of CHS-induced skin inflammation [7]. Thus, upregulation of endogenous VEGF-A in the inflamed skin of WT mice may partially explain why no major differences were observed between the experiments performed in WT as compared to K14-VEGF-A-tg mice.

Our data suggest that the phenotype of DCs present in LNs draining CHS-inflamed skin likely accounts for the observed differences in immune priming: DCs isolated from LNs draining inflamed skin were more potent in inducing T cell proliferation and effector cytokine production in in vitro co-culture experiments, as compared to DCs from control LNs (**Figure 4** **, Figure S4 in File S1**). Furthermore, they expressed higher levels of co-stimulatory and MHC molecules and of IL-12/23-p40 (**Figure 5** **&** **6** **, Figure S5&S7 in File S1**). The more mature DC phenotype in LNs draining sites of oxazolone-induced skin inflammation may have been caused by a CHS-induced increase in the production of pro-inflammatory cytokines and by an oxazolone-induced release of DAMPs, resulting in the upregulation of co-stimulatory molecules and cytokine production by DCs [4]–[6], [30]. In fact, because of the latter properties, haptens have already been specifically used as adjuvants to enhance immunity towards protein antigens [31]. In our study CHS-induced upregulation of co-stimulatory molecules and MHC molecules was not restricted to the CD11c^+^ I-A/I-E^hi^ DCs, which preferentially arrive in dLNs via afferent lymphatics. Upregulation was also observed in CD11c^+^ I-A/I-E^int/lo^ cells, which form part of the LN-resident DC pool (**Figure S6 in File S1**). The latter may also participate in the induction of adaptive immunity in dLNs by presenting antigen that has drained to the node via the lymphatic vessel network [32]. However, we presently do not know whether the migratory or the LN-resident DC pool is more important for immune induction in our model.

Interestingly, most co-stimulatory molecules and MHC molecules were strikingly upregulated in DCs present in skin-draining LNs at the DAY 2 time point, whereas a much less pronounced upregulation was detected at the DAY 9 time point. This was observed in both the K14-VEGF-A and in the WT CHS model studied (**Figure 5** and **Figure S5 in File S1**). The reason for this is presently unclear, but argues for clear differences in the inflammatory milieu present in DAY 2 and DAY 9 CHS-induced skin inflammation. In line with this conclusion our analysis of TNFα, IFNγ or IL17 expression in the inflamed ears of K14-VEGF-A-tg mice revealed significant differences in cytokine levels between the DAY 2 and the DAY 9 time points (**Figure 1C–E**). Surprisingly, despite significant differences in co-stimulatory and MHC molecule expression, DCs present in LNs draining DAY 2- and DAY 9-inflamed skin shared a similar capacity to induce T cell activation (**Figure 4** and **Figure S4 in File S1**). IL-12/23-p40 was the only DC-expressed factor analyzed, which we found to be similarly upregulated at both time-points of inflammation and thus to correlate best with our findings (**Figure 6** and **Figure S8 in File S1**). Our experiments revealed that in WT mice blockade of IL-12/23-p40 at the time of T cell priming with OVA resulted in an attenuation of inflammation-induced DTH and anti-OVA antibody responses (**Figure 7**). Since IL-12/23-p40 forms a subunit of both IL-12 and of IL-23 [33] it is presently unclear which of these cytokines accounted for the observed reduction in immune priming in the context of CHS-induced skin inflammation. Notably, IL-12/23-p40 has already previously been implicated in the induction of DTH responses: Specifically, studies in IL-12/23-p40^−/−^ mice have shown that the induction of a DTH response upon immunization with protein antigen and CFA was strongly reduced in absence of IL-12/23-p40 [34], [35]. Moreover, humoral immunity was impaired in IL-12/23-p40^−/−^ mice immunized with protein and CFA [35]. Our new data show that the adjuvanticity of oxazolone-induced skin inflammation also partially depends on IL-12/23-p40 expression.

Although our data suggest that DCs account for the enhanced immune induction observed in the context of CHS-induced skin inflammation, we cannot rule out that other cell types also contribute to this process. Besides DCs, neutrophils and particularly macrophages are important sources of IL-12/23-p40 [36]. All three cell types participate in CHS responses [1], [2], [37] and therefore could be potential sources of IL-12/23-p40. At the same time, it is clear that the immune-enhancing activity of oxazolone-induced skin inflammation cannot exclusively be explained by IL-12/23p40. In fact, MHC I and II molecules, CD40 and also the inhibitory molecule PD-L1 were significantly upregulated at both the DAY 2 and the DAY 9 time points (**Figure 5D–G** and **Figure S5D–G in File S1**). Although the upregulation of MHC molecules and of the co-stimulatory molecule CD40 was much less profound at the DAY 9 as compared to the DAY 2 time point (**Figure 5E–G** and **Figure S5E–G in File S1**), it is still conceivable that these molecules also contribute to enhancing adaptive immunity in the context of CHS. Interestingly, CD40 and IL-12/23-p40 expression in DCs is thought to be directly dependent, since CD40 ligation was shown to induce IL-12/23-p40 upregulation [38], [39].

Notably, immunization with OVA alone into uninflamed control skin (CTR) was capable of inducing a low anti-OVA IgG response (**Figure 3G**), suggesting that the OVA or the injection technique used were not completely free of endotoxins. This hypothesis is further supported by the fact that a mild ear swelling response was also observed upon induction of an OVA-specific DTH response in CTR mice (**Figure 3B**). Thus, the parameters measured in the CTR group (**Figure 2** **,** **Figure 3** **,** **Figure 7**) likely did not reflect completely uninflamed, baseline conditions. Nevertheless, the adaptive immune response observed upon immunization with OVA in DAY 2- or DAY 9-inflamed skin was much more pronounced and comparable to the response induced upon immunization in presence of conventional adjuvants (**Figure 2** **,** **Figure 3**).

Overall, our data indicate that in the context of CHS-induced inflammation it is not so much the functional state of the lymphatic network but more the adjuvanticity of the inflamed tissue, which impacts the induction of an adaptive immune response. It will be interesting to explore in future studies whether this is a specific feature of CHS-induced inflammation or whether these findings also hold true for other inflammatory conditions. Emerging data from studies performed in animal models and in human patients indicate that lymphatic drainage may be reduced in various chronic inflammatory and autoimmune disorders, such as in rheumatoid arthritis [40], psoriasis [11], [41] or in inflammatory bowel disease [42], [43]. At the same time tissue biopsies have shown that DCs present in psoriatic lesions [44], [45], inflamed synovium [46] or inflamed intestine [47] display an activated phenotype. The relationship between lymphatic function and the induction of adaptive immunity in the context of these inflammatory conditions warrants further investigation.

## Materials and Methods

### Ethics statement

Animal experiments were performed in strict accordance to the Swiss Federal Regulations. The protocol was approved by the Cantonal Veterinary Office Zurich (licence number: 117/2011). All efforts were made to minimize suffering and the number of mice needed to reach statistical significance and experimental reproducibility.

### Mice

WT FVB or BALB/c mice were purchased from Janvier, Genest-Saint-Isle, France, or from Charles River Laboratories, Sulzbach, Germany. Hemizygous K14-VEGF-A transgenic (K14-VEGF-A-tg) mice were generated by crossing homozygous K14-VEGF-A-tg mice [13] with WT FVB or BALB/c mice. TCR transgenic DO11.10 mice [25] were from The Jackson Laboratories (Bar Harbor, ME, USA). All animals were bred and housed in our animal facility, and experiments were approved by the Cantonal Veterinary Office Zurich (licence number: 117/2011).

### Induction of a CHS response to oxazolone

Hemizigous K14-VEGF-A-tg mice were anesthetized by intraperitoneal administration of medetomidine (1 mg/ml) and ketamine (75 mg/kg) and sensitized by topical application of 2% oxazolone (4-ethoxymethylene-2-phenyl-2-oxazoline-5-1, Sigma, St. Louis, MO) in acetone/olive oil (4∶1 vol/vol) on the shaved abdomen (50 µl) and on each paw (5 µl). Five days later mice were challenged by topical application of 10 µl of 1% oxazolone on each side of the ear. Experiments were performed 2 or 9 days after challenge (DAY 2 and DAY 9 time point). In some experiments, a CHS response to oxazolone was induced in the ears of WT BALB/c mice and maintained by repeatedly challenging the ears with 1% oxazolone on day 2 and 5 after onset of inflammation (as depicted in Figure 2A). Animals were analyzed 2 or 9 days after the first challenge (i.e. DAY 2 and DAY 9).

### FACS analysis of ear and LN single-cell suspensions

Ear single-cell suspensions were generated by digestion with collagenase IV (Invitrogen, Basel, Switzerland) followed by passage through a 40 µm cell strainer (BD Biosciences, Basel, Switzerland) as previously described [48], [49]. Cell suspensions were stained with hamster-anti-mouse CD11c-APC (clone: N418), rat-anti-mouse I-A/I-E-PerCP (clone: M5/114.15.2), rat-anti-mouse CD4-PE (clone: GK1.5), rat-anti-mouse CD8-FITC (clone: 53–6.7), rat-anti-mouse CD3-APC (clone: 145-2C11), rat-anti-mouse CD11b-FITC (clone: M1/70), rat-anti-mouse CD45-PerCP (clone: 30-F11) and rat-anti-mouse Gr1-PE (clone: RB6-8C5) (all from BioLegend, San Diego, CA, USA). LN single-cell suspensions were generated by passage through a 40 µm cell strainer. Cell suspensions were stained for hamster-anti-mouse CD11c-APC (clone: N418), rat-anti-mouse I-A/I-E-PerCP (clone: M5/114.15.2), rat-anti-mouse CD40-PE (clone: 3/23), hamster-anti-mouse CD80-FITC (clone: 16-10A1), rat-anti-mouse CD86-PE (clone: GL-1), rat-anti-mouse PD-L1-PE (clone: 10F.9G2), mouse-anti-mouse H-2K^q^-FITC (clone: KH114), mouse-anti-mouse H-2K^d^-FITC (clone: SF1-1.1), mouse-anti-mouse I-A^q^-FITC (clone: KH116) mouse-anti-mouse I-A^d-^FITC (clone: 39-10-8) biotinylated rat-anti-mouse OX40L (clone: RM134L) (all from Biolgend), corresponding isotype controls and streptavidin-PE (Invitrogen). All FACS analyses were performed on a BD FACSCanto using FACSDiva software (BD Biosciences). Data were analyzed with FlowJo software (Version 8.7.1; Treestar, Ashland, TN, USA). In some experiments cells were quantified using counting beads (Invitrogen).

### Intracellular staining of IL-12/23p40

For intracellular cytokine staining, single cell suspensions were stained with hamster-anti-mouse CD11c-APC (clone: N418, BioLegend) and subsequently fixed and permeabilized using the BD Cytofix/Cytoperm Kit (BD Biosciences). Intracellular staining was performed using rat-anti-mouse IL-12/23p40-PE (clone: C15.6) or a rat-IgG_1_-PE (both from BioLegend).

### Quantification of other cytokines

Cytokines were quantified in tissue protein extracts and cell culture supernatants using the FlowCytomix kit (eBioscience, Vienna, Austria) according to the manufacturer's protocol. For quantification of cytokines in the ear tissue, ears were incubated in a protein extraction buffer containing 150 nM NaCl, 50 mM Tris and protease inhibitor cocktail (Roche Diagnostics, Rotkreuz, Switzerland). 5-mm steel beads (Qiagen, Hilden, Germany) were added to the samples, and the homogenization process was performed using a Qiagen tissue lyzer (4×1 min, 4°C, 30 Hz). Homogenized tissues were then centrifuged for 5 min at full speed and cytokines were quantified in the supernatant.

### Sorting of DCs

K14-VEGF-A-tg mice (MHC-II I-A^dq^, for detailed description see supplemental Methods) with uninflamed (CTR), or DAY 2- and Day 9-CHS-inflamed ear skin were sacrificed and the draining auricular LNs harvested. Single-cell suspensions were generated by passage through a 40 µm cell strainer (BD Biosciences). Samples were stained with hamster-anti-mouse CD11c-APC (clone: N418) and rat-anti-mouse I-A/I-E-PerCP (clone: M5/114.15.2) (both from BioLegend). And CD11c^+^ I-A/I-E^+^ DCs were isolated on a FACSAria Cell Sorter (BD Biosciences), sorted into fresh medium and used in co-culture assays with DO11.10-tg CD4^+^ T cells.

### In vitro CD4^+^ T cell activation

Spleen and LNs of DO11.10 mice were harvested, digested in RPMI-1640 medium (GIBCO, Paisley, UK) containing liberase CI (0.42 mg/ml (1.67 U/ml), Roche) and DNase 1 (0.2 mg/ml (400 U/ml), Roche) and passed through a 40 µm cell strainer (BD Biosciences). Single-cell suspensions were incubated for 20 min with anti-CD4^+^-coupled magnetic beads and isolated using a MACS column and separator (all from Miltenyi Biotech, Gladbach, Germany). Isolated CD4^+^ T cells (purity > 90%) were stained with 2 µM CFSE (Invitrogen) for 12 min at 37°. Subsequently, T cells were co-cultured for 3 days with FACS-sorted DCs, at a T cell:DC ratio of 5∶1, in presence of 100 µM OVA peptide (residues 323-339, GenScript, Piscataway, NJ, USA). Co-cultures were performed in RPMI-1640 medium (GIBCO) supplemented with 10% fetal bovine serum (FBS; GIBCO), 50 µM β-mercaptoethanol (Sigma), 15 mM HEPES (GIBCO), 1 mM Na-pyruvate (GIBCO), antibiotic/antimycotic solution (Fluka, Buchs, Switzerland) and 2 mM L-glutamine (Fluka).

### FITC painting

Mice were painted on the ears with fluorescein isothiocyanate (FITC - 0.5%, Thermo Scientific, Wohlen, Switzerland) dissolved in acetone/dibutylphtalae (1∶1 vol/vol - Sigma) as previously described [48], [50]. 18 hours later mice were sacrificed and ear draining auricular LNs were harvested and passed through a 40 µm cell strainer (BD Biosciences). Total cell numbers were counted, cells stained with hamster anti-mouse CD11c-APC (clone: N418), and rat anti-mouse I-A/I-E-PerCP (clone: M5/114.15.2) (both from Biolegend) and samples acquired on a BD FACSCanto (BD Biosciences). Offline analysis was performed with FlowJo (Treestar). The number of FITC^+^CD11c^+^I-A/I-E^+^ DCs was determined by multiplying the number of total LN cells by the fraction of FITC^+^CD11c^+^I-A/I-E^+^ cells obtained by FACS analysis.

### Induction of a DTH response to ovalbumin (OVA)

Mice were first sensitized in the right ear by injection of 100 µg of OVA protein (Labforce AG, Nunningen, Switzerland) in 10 µl of PBS into the ear skin. As a positive control mice were either injected with a solution containing 100 µg of OVA protein emulsified in Complete Freund's Adjuvant (CFA, Sigma), or containing 100 µg of OVA and 5 µg of CpG oligonucleotide [51] (TCCATGACGTTCCTGATGCT - Microsynth Balgach, Switzerland). Seven days later mice were anesthetized with isoflurane and the ear thickness of the untouched, left ear was measured using a caliper. Subsequently, mice were challenged by injection of 50 µg of OVA in the left ear skin. 48 hours after the challenge the ear thickness of the left ear was again measured, and mice were sacrificed for further analysis of the ears. For leukocyte quantification the ventral and dorsal sheets of the ear were separated along the cartilage using forceps. The two leaflets were incubated at 37°C, dermal side down, in a well of a 24-well plate containing RPMI-1640 media (Gibco) supplemented with 10% FBS (GIBCO) and antibiotic/antimycotic solution (Fluka). The leukocyte populations emigrating spontaneously over 14 hours from the ear explants were stained with rat-anti-mouse CD4-PE (clone: GK1.5), rat-anti-mouse CD8-FITC (clone: 53–6.7), rat-anti-mouse CD3-APC (clone: 145-2C11), rat-anti-mouse CD11b-FITC (clone: M1/70), rat-anti-mouse CD45-PerCP (clone: 30-F11) and rat-anti-mouse Gr1-PE (clone: RB6-8C5) (all from BioLegend). Cells were quantified by FACS analysis using counting beads.

### IL-12/23p40 blockade

Mice were treated by intraperitoneal injection of 100 µg of rat-anti-mouse IL-12/23p40 antibody (clone: C17.8, Biolegend) or of 100 µg of rat IgG2a isotype control (Biolegend) once. One day later a DTH response to OVA was induced by sensitizing mice as described in the previous section and in **Figure 7A**. Ten days later mice were anesthetized with isoflurane and the ear thickness of the untouched, left ear was measured using a caliper. Subsequently, mice were challenged by injection of 50 µg of OVA in the left ear skin. 48 hours after the challenge the ear thickness was again measured, and mice were sacrificed for further analysis.

### Serum IgG quantification

Blood was collected by cardiac puncture, incubated for 5 min at 37°C, centrifuged and the serum collected. Serum IgG quantification was performed by ELISA, as described in Methods S1 in File S1.

### Assessment of lymphatic drainage function

3 µl of an Evans Blue dye solution (10 mg/ml in PBS, Fluka) were injected into the ear of anesthetized mice using a Hamilton syringe (Hamilton, Bonaduz, Switzerland) and pictures of the ears were taken using a Canon DS126191 camera. 16 hours later, mice were sacrificed and the Evans Blue dye was extracted from the ears by incubation for 3 days in formamide (Fluka) at room temperature. The background-subtracted absorbance was measured on an Infinite M200 microplate reader by measuring the wavelength at 620 nm and 740 nm. The concentration of dye in the extracts was calculated using a standard curve of Evans Blue in formamide.

### Statistical analysis

Data were analyzed for normal distribution using the Kolmogorov-Smirnov test and normally distributed data were analyzed using the one-way ANOVA followed by post hoc analysis. Data sets, which were not normally distributed, were analyzed using the Kruskal-Wallis test followed by post hoc analysis. Results are represented as mean ± standard error of the mean (SEM). Differences were considered statistically significant when p<0.05. Statistical analysis was performed with Prism 5 (GraphPad, La Jolla, CA, USA).

## Supporting Information

File S1 **This file includes Figures S1 to S8 and Methods S1.**
(PDF)
