# Supplementary material for: Oxazolone-Induced Contact Hypersensitivity Reduces Lymphatic Drainage but Enhances the Induction of Adaptive Immunity
Source: PLoS One. 2014 Jun 9;9(6):e99297. doi: 10.1371/journal.pone.0099297 (PMC4050031; doi:10.1371/journal.pone.0099297)
Supplement: File S1 — This file includes Figures S1 to S8 and Methods S1. (PDF) [file pone.0099297.s001.pdf]

Supplemental Methods:

Ear explant emigration assay

Mice were sacrificed and the ears harvested. The ventral and dorsal sheets of the ear were separated along the cartilage using forceps. The two leaflets were incubated at 37°C, dermal side down, in wells of a 24-well plate containing RPMI-1640 media (Gibco) supplemented with CCL21 (75 nM, Peprotech, Rocky Hill, NJ, USA) and antibiotics. The leukocyte populations emigrating spontaneously over 14 hours from the ear explants were stained with hamster anti-mouse CD11c-APC, rat anti-mouse I-A/I-E-PerCP (both from BioLegend). Cells were quantified by FACS analysis using counting beads.

Induction of a CHS response to FITC

Anesthetized mice were sensitized by topically applying a 2% FITC solution (20 mg/ml) in acetone/dibutylphtalate (1:1 vol/vol) on each side of the right ear (20 µl/side). Five days later mice were anesthetized and the ear thickness of the untouched, left ear was measured using a caliper. Subsequently, mice were challenged by topical application of a 0.5% FITC (5 mg/ml) solution in acetone/dibutylphtalate on the left ear. 48 hours later the ear thickness of the left ear was again measured and mice were sacrificed for further analysis.

Serum IgG quantification

For the quantification of IgG, a maxisorb 96 well plate (Nunc International, Naperville, IL, USA) was coated with 5 µg/ml of OVA (Labforce AG). After blocking with 10% FBS (Gibco) in PBS, serum samples were applied at different dilutions. IgG levels were detected with a horseradish peroxidase (HRP)-coupled rabbit anti-mouse IgG antibody (Zymed Laboratories, SanFrancisco, CA). Alternatively, IgG_1 and IgG_{2a} levels were detected with biotinylated antibodies directed against mouse IgG_1 (clone: RMG1-1, BioLegend - 0.5 µg/ml) or mouse IgG_{2a} (clone: RMG2a-62, BioLegend – 0.5 µg/ml), followed by incubation with streptavidin- HRP (Invitrogen). The ELISA was developed using 2,2'–Azino-bis(3-ethylbenzothiazoline-6-sulfonic acid) (ABTS,

Sigma) substrate and the reaction stopped with 1% SDS. The absorbance at 405 nm was measured with a plate reader (BioRad, Hercules, CA, USA). Values measured for the treatment groups were normalized by subtracting the absorbance value measured in serum of untreated control mice.

Generation of K14-VEGF-A-tg mice with MHC I-A^{dq}

Homozygous K14-VEGF-A-tg mice on an FVB background (MHC II I-A^{q}) were crossed with BALB/c mice (MHC II I-A^{d}) and the resulting hemizygous K14-VEGF-A-tg F1 generation (MHC II I-A^{dq}) was used for subsequent experiments (i.e. the experiment described in **Figure S4**). Pilot experiments confirmed that OVA-pulsed, bone-marrow-derived DCs generated from mice of the F1 generation were capable of inducing T-cell proliferation and cytokine secretion in I-A^{d} restricted $CD4^{+}$ T cells isolated from T-cell receptor (TCR) transgenic DO11.10 mice [1] (data not shown).

Supplemental References:

1. Murphy KM, Heimberger AB, Loh AY (1990) Induction by antigen of intrathymic apoptosis of $CD4^{+}CD8^{+}$ TCR^{lo} thymocytes in vivo. Science 250: 1720-1723.

Supplemental Figure Legends:

Supplemental Figure 1. DCs present in inflamed skin display a transient decrease in their ability to emigrate form CHS-inflamed ear skin no increase in their intrinsic migratory capacity. The intrinsic migratory capacity of DCs present in DAY 2- and DAY 9-inflamed or control ears was studied in an ear emigration assay. Ear halves were placed into

medium containing CCL21 and the number of $CD11c^+I\text{-}A/I\text{-}E^+$ DCs found in the medium or retained in the ear tissue was quantified 16 hours later. (**A**) Representative FACS plots documenting the gating scheme. (**B**) Quantification of emigrated DCs found in the culture medium. (**C**) Quantification of DCs isolated from the ear tissue. (**D**) Percentages of retained as compared to emigrated DCs. Representative data from 1 out of 3 similar experiments (n = 5 mice / group) are shown. * $p<0.05$.

Supplemental Figure 2. Quantification of leukocytes in ear skin after induction of a DTH response to OVA. FACS analysis was performed on single-cell suspensions of the DTH-challenged left ears to quantify leukocyte infiltration. The FACS plots outline the staining and gating strategy used to quantify (**A**) total leukocytes ($CD45^+$), (**B**) neutrophils ($CD11b^+Gr1^+$), and (**C**) $CD4^+$ T cells ($CD4^+CD3^+CD45^+$) and $CD8^+$ T cells ($CD8^+CD3^+CD45^+$).

Supplemental Figure 3. Skin inflammation facilitates the induction of a CHS response to FITC in dLNs of K14-VEGF-A-tg mice. (**A**) Schematic representation of the experiment: K14-VEGF-A-tg mice were grouped into a control (CTR) group, a DAY 2 and a DAY 9 group. On day -14 and day -7, mice from the DAY 9 group or the DAY 2 group, respectively, were sensitized by application of oxazolone (OXA) onto the belly and paws (indicated as OXA-SENS). 5 days later (day -9 or day -2) a CHS response was induced, by challenging mice from the DAY 9 and DAY 2 groups with oxazolone on the right ear (indicated as OXA-CHALL). On day 0, corresponding to 2 days (DAY 2 group) or 9 days (DAY 9 group) after the onset of inflammation (marked as a red bar), mice in all treatment groups were sensitized by applying FITC onto the right ear. 5 days later, mice were challenged by applying FITC onto the left, uninflamed ear. The strength of the CHS response induced in the left ear was analyzed 2 days later (day 7). (**B**) The ear swelling response in the left ear was significantly stronger when mice had been primed by FITC application onto the DAY 2- or DAY 9-inflamed right ear. The Δ Ear thickness is shown, defined as the difference between the ear thickness measured at baseline and the ear thickness measured two days after challenge. (**C-E**) FACS analysis was performed

on single-cell suspensions generated from the CHS-challenged left ear to quantify leukocyte infiltration. Analysis revealed that FITC sensitization in DAY 9-inflamed skin induced a significantly higher degree of leukocyte infiltration as compared to sensitization in uninflamed control skin. Quantification of (**C**) total leukocytes ($CD45^+$), (**D**) neutrophils ($CD11b^+$,$Gr1^+$) and (**E**) $CD3^+$ cells. The FACS plots outline the staining and gating strategy. Representative data from 1 out of 3 similar experiments (n = 5 mice / group) are shown. * $p<0.05$; ** $p<0.01$; *** $p<0.001$.

Supplemental Figure 4. DCs isolated from LNs draining CHS-inflamed skin of K14-VEGF-A-tg mice are more potent inducers of T cell activation in vitro. $CD11c^+$ DCs were FACS-sorted from the auricular LNs of K14-VEGF-A-tg mice with uninflamed (CTR) or DAY 2- or DAY 9-inflamed ear skin. Subsequently, CFSE-labeled TCR transgenic $CD4^+$ T cells isolated from D011.10 mice were incubated for 3 days with DCs and OVA peptide at a T cell: DC ratio of 5:1. CFSE dilution assays revealed that DCs isolated from LNs draining inflamed ears (both DAY 2 and DAY 9) were significantly more potent in inducing T cell proliferation than DCs isolated from control LNs. (**A**) Representative FACS plots showing CFSE dilution in dividing T cells. Numbers indicate the percentage of gated cells. (**B**) Quantification of the percentage of proliferating T cells. Significantly higher levels of (**C**) IFNγ, (**D**) IL-17 and (**E**) IL-4 were detected in co-cultures containing DCs isolated from LNs draining DAY 2- or DAY 9-inflamed skin. Representative data from 1 out of 3 similar experiments (n = 3) per condition are shown. * $p<0.05$.

Supplemental Figure 5. DCs in LNs draining sites of skin inflammation in K14-VEGF-A-tg display changes in their expression of co-stimulatory and MHC molecules. FACS analysis was performed on $CD11c^+$ DCs present in auricular LNs draining DAY 2- and DAY 9-inflamed or uninflamed control (CTR) skin of K14-VEGF-A-tg mice. (**A-F**) Analysis of the expression levels of co-stimulatory and MHC molecules. The upper panel shows representative FACS plots. Blue line: CTR; green line: DAY 2: red line: DAY 9; filled histogram: isotype control. To

reduce complexity, only one out of three similar isotype controls is shown. The lower graph shows the Δ MFI (defined as the MFI of the specific staining – the MFI of the isotype control staining) values measured for each condition (n = 3 mice / group). (**A**) CD80, (**B**) CD86, (**C**) OX40L, (**D**) PD-L1, (**E**) CD40, (**F**) H-2K^q and (**G**) I-A^q. Representative data from 1 out of 3 similar experiments are shown. *p < 0.05; **p < 0.01; ***p < 0.001.

Supplemental Figure 6. Lymph-borne DCs and blood-borne DCs found in skin-draining LNs respond similarly to skin inflammation. FACS analysis was performed on CD11c$^+$ I-A/I-E^{hi} (lymph-borne) and CD11c$^+$,I-A/I-E$^{low/int}$ (blood-borne) DCs present in auricular LNs draining day DAY 2- or DAY 9-inflamed or uninflamed skin of K14-VEGF-A-tg mice. The first two panels of each row show a representative FACS plot and the quantification of each molecule in CD11c$^+$I-A/I-E$^{low/int}$ (blood-borne) DCs. The last two panels of each row show the representative FACS plot and the quantification of each molecule in CD11c$^+$I-A/I-E^{hi} (lymph-borne) DCs. FACS plots: Blue line: CTR; green line: DAY 2; red line: Day 9; filled histogram; isotype control. The quantifications show the Δ MFI (defined as the MFI of the specific staining – the MFI of the isotype control staining) values measured for each condition. (**A**) CD80, (**B**) CD86, (**C**) OX40L, (**D**) PD-L1, (**E**) CD40 (**F**) H-2K^q and (**G**) I-A^q. Representative data from 1 out of 3 similar experiments (n = 3 mice / group) are shown. *p < 0.05; **p < 0.01; ***p < 0.001.

Supplemental Figure 7. Analysis of migratory and resident DCs in LNs draining DAY 2- and DAY 9-inflamed or uninflamed control (CTR) skin. FACS analysis was performed to evaluate CD11c$^+$ I-A/I-E^{hi} (migratory, lymph-borne) and CD11c$^+$,I-A/I-E$^{low/int}$ (resident, blood-borne) DCs present in auricular LNs draining day DAY 2- or DAY 9-inflamed or uninflamed skin of K14-VEGF-A-tg mice. The plot shows the percentage of I-A/I-E^{hi} (lymph-borne) and I-A/I-E$^{low/int}$ (blood-borne) cells amongst all CD11c$^+$ cells. Pooled data from three experiments are shown (total n = 10-11 mice / group).

Supplemental Figure 8. DCs in LNs draining sites of skin inflammation in K14-VEGF-A-tg mice upregulate IL-12/23p40 expression. Intracellular FACS analysis of IL-12/23-p40 in $CD11c^+$ DCs present in LNs draining uninflamed control (CTR) or DAY 2- or DAY 9-inflamed ear skin of K14-VEGF-A-tg mice. (**A**) Representative FACS plots of the different groups are shown. (**B**) The MFI of IL-12/23-p40 staining on $CD11c^+$ DCs was significantly higher in DCs present in LNs draining DAY 2- and DAY 9-inflamed ear skin as compared to DCs in control LNs (**C**) Furthermore, a greater percentage of DCs in inflammation-dLNs stained positive for IL-12/23-p40 expression as compared to DCs in control LNs. Representative data from 1 out of 3 similar experiment (n = 3 mice / group) are shown. *$p < 0.05$; **$p < 0.01$; ***$p < 0.001$.

Figure S1

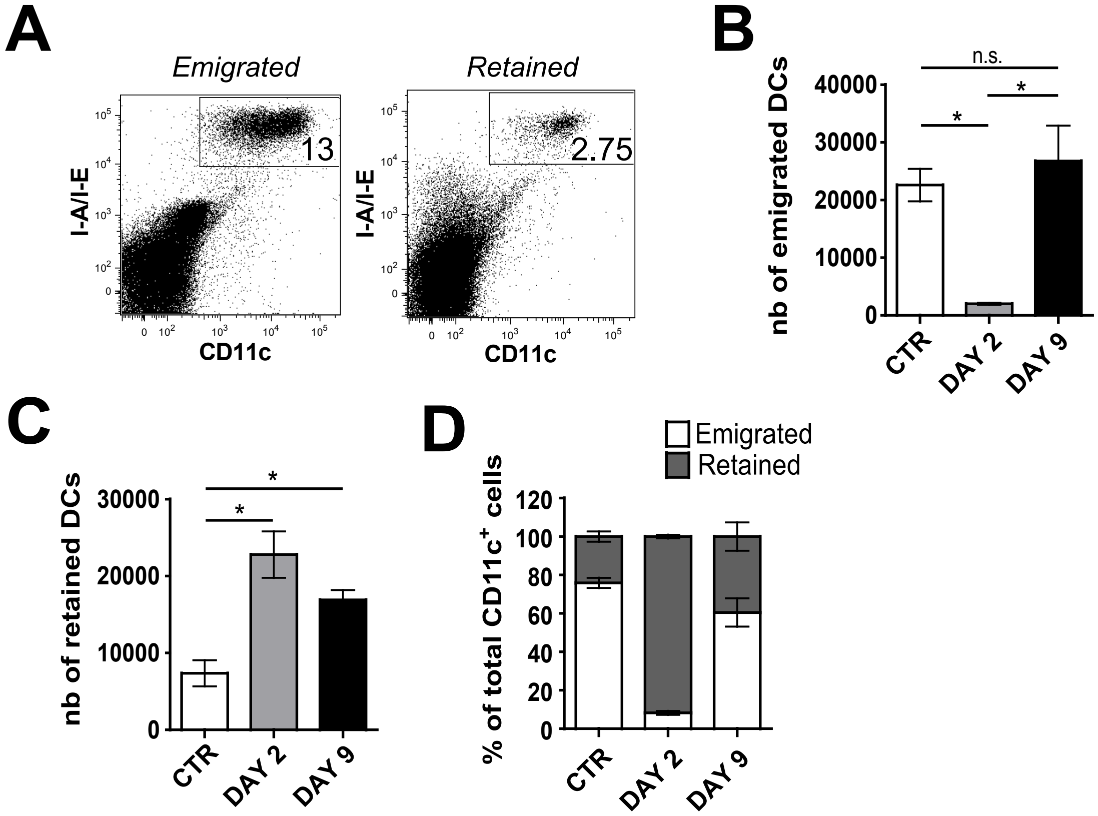

A
Emigrated
Retained
I-A/I-E
CD11c
13
2.75
B
n.s.
*
*
nb of emigrated DCs
40000
30000
20000
10000
0
CTR
DAY 2
DAY 9
C
*
*
nb of retained DCs
30000
20000
10000
0
CTR
DAY 2
DAY 9
D
Emigrated
Retained
% of total CD11c+ cells
120
100
80
60
40
20
0
CTR
DAY 2
DAY 9

Figure S2

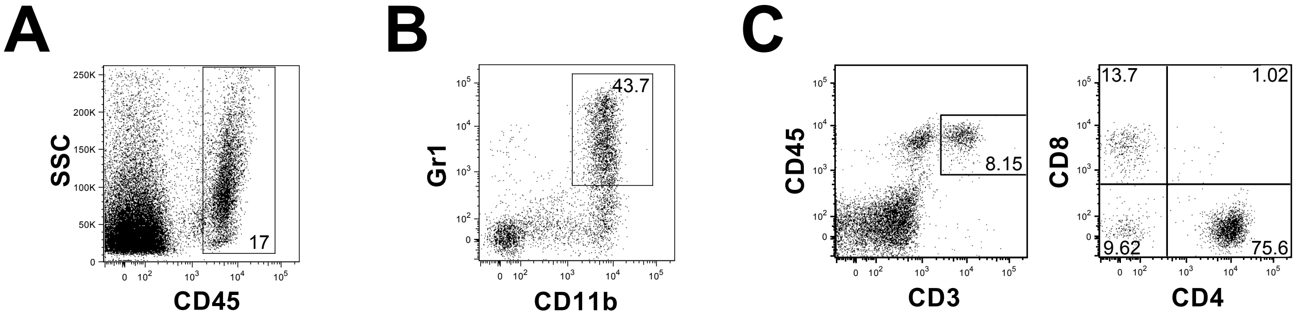
A
SSC
CD45
17
B
Gr1
CD11b
43.7
C
CD45
CD3
8.15
CD8
CD4
13.7
1.02
9.62
75.6

Figure S3

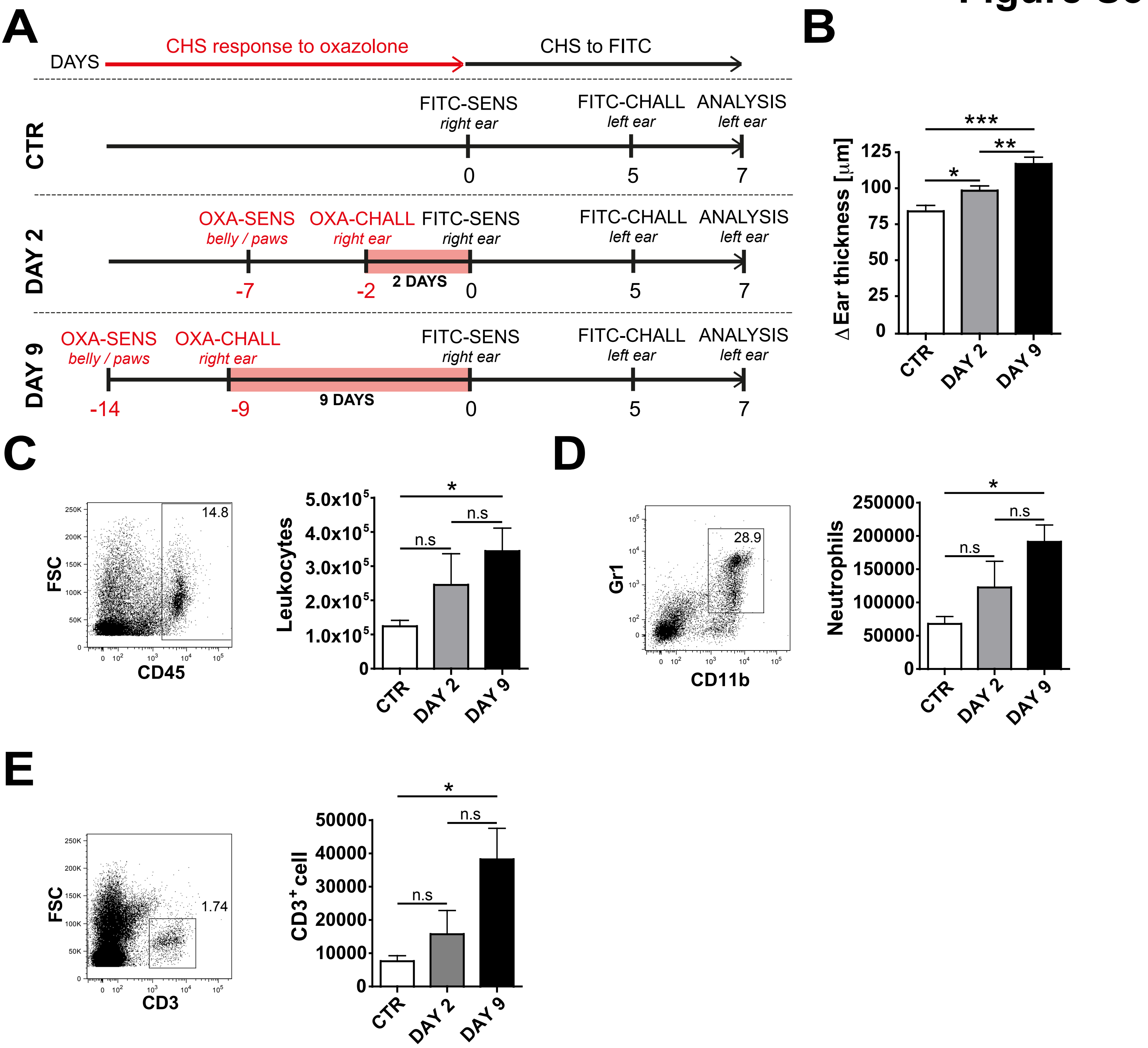
A
DAYS
CHS response to oxazolone
CHS to FITC
CTR
FITC-SENS
right ear
FITC-CHALL
left ear
ANALYSIS
left ear
0
5
7
DAY 2
OXA-SENS
belly / paws
OXA-CHALL
right ear
-7
-2
2 DAYS
DAY 9
-14
-9
9 DAYS
B
Δ Ear thickness [μm]
CTR
DAY 2
DAY 9
C
FSC
CD45
14.8
Leukocytes
n.s
D
Gr1
CD11b
28.9
Neutrophils
E
CD3
1.74
CD3+ cell

Figure S4

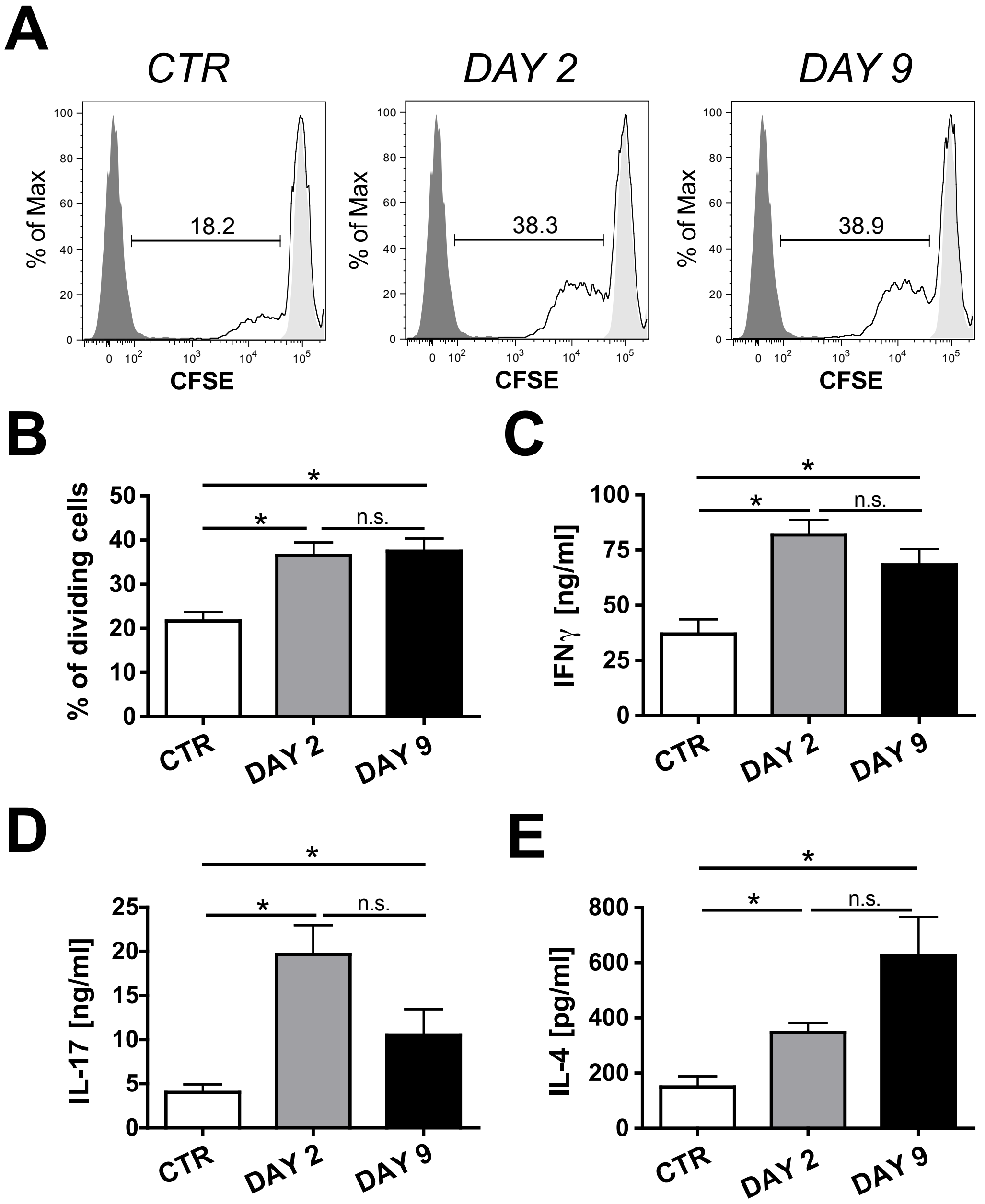
A
CTR
DAY 2
DAY 9
% of Max
CFSE
18.2
38.3
38.9
B
% of dividing cells
C
IFNγ [ng/ml]
D
IL-17 [ng/ml]
E
IL-4 [pg/ml]
CTR
DAY 2
DAY 9
*
n.s.

Figure S5

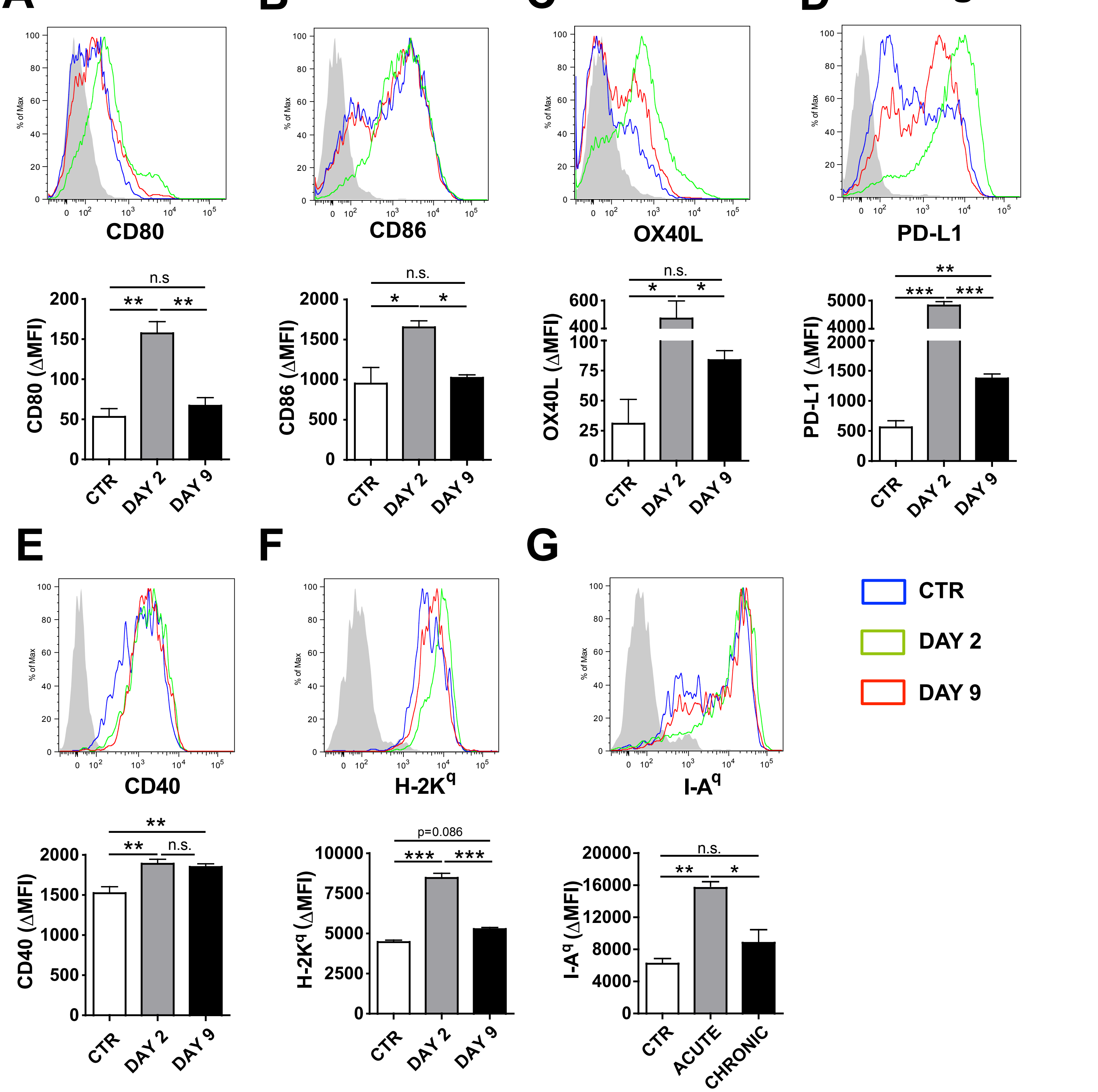
A
B
C
D
CD80
CD86
OX40L
PD-L1
% of Max
CD80 (ΔMFI)
CD86 (ΔMFI)
OX40L (ΔMFI)
PD-L1 (ΔMFI)
n.s
n.s.
CTR
DAY 2
DAY 9
E
F
G
CD40
H-2Kq
I-Aq
CD40 (ΔMFI)
H-2Kq (ΔMFI)
I-Aq (ΔMFI)
p=0.086
ACUTE
CHRONIC

Figure S6

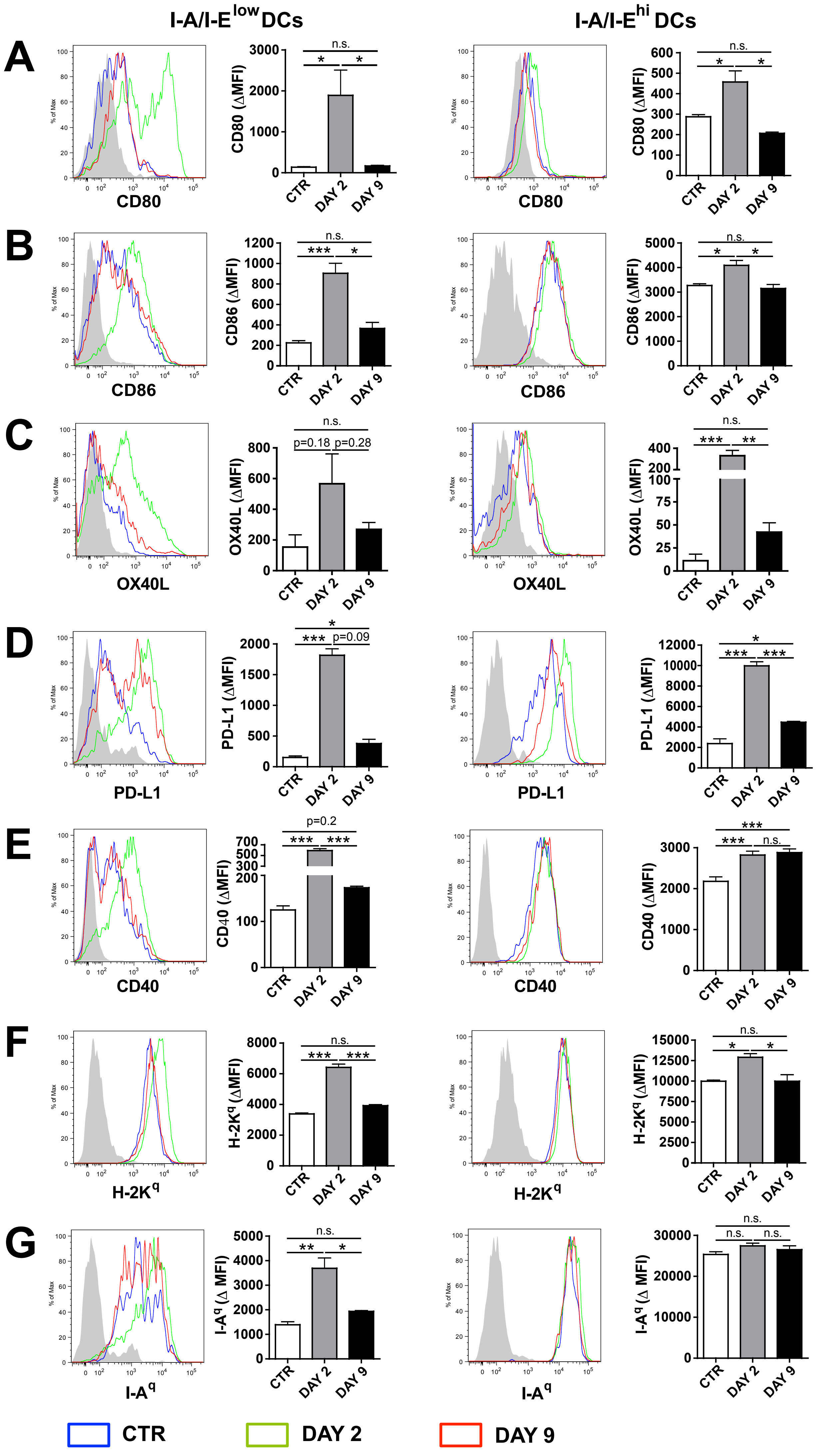

I-A/I-E^low DCs
I-A/I-E^hi DCs
A
CD80
CD80 (ΔMFI)
n.s.
*
CTR
DAY 2
DAY 9
B
CD86
CD86 (ΔMFI)
***
C
OX40L
OX40L (ΔMFI)
p=0.18
p=0.28
**
D
PD-L1
PD-L1 (ΔMFI)
p=0.09
E
CD40
CD40 (ΔMFI)
p=0.2
F
H-2Kq
H-2Kq (ΔMFI)
G
I-Aq
I-Aq (Δ MFI)
% of Max

Figure S7

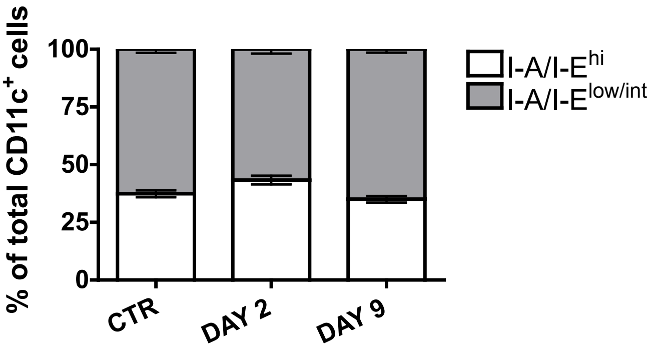

% of total CD11c+ cells
100
75
50
25
0
CTR
DAY 2
DAY 9
I-A/I-Ehi
I-A/I-Elow/int

Figure S8

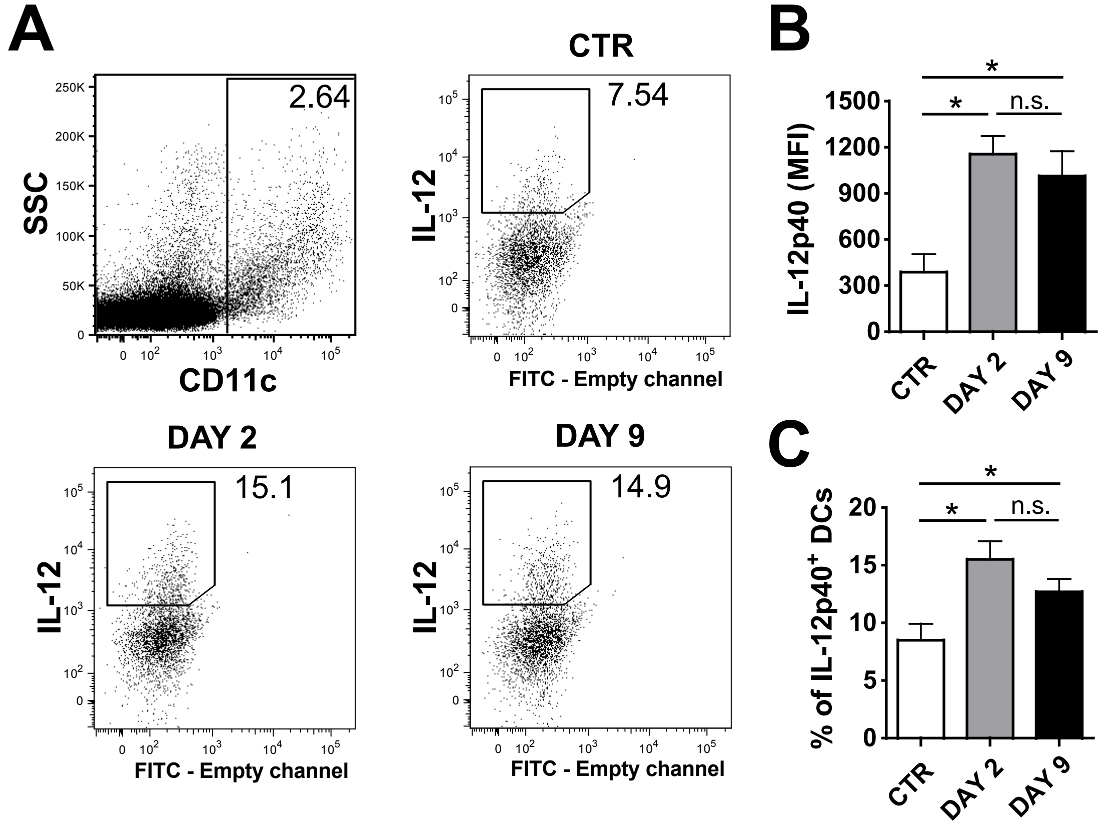

A
B
C
SSC
CD11c
2.64
CTR
7.54
IL-12
FITC - Empty channel
DAY 2
15.1
DAY 9
14.9
IL-12p40 (MFI)
1500
1200
900
600
300
0
*
n.s.
CTR
DAY 2
DAY 9
% of IL-12p40+ DCs
20
15
10
5
0
